# Separating Material Reactivity from Residual Oxygen in TGA–TOF MS of Natural and Artificial Graphite Anodes

**DOI:** 10.3390/ma19214550

**Published:** 2026-10-28

**Authors:** Hee-Jin Yoo, Minsun Kim, Young Hwan Kim, Woo Jin Jeon, Byung Gwun Jin, Jooyeon Oh, Hyun Sik Kim, Yangsun Kim, Young-Kwan Kim, Han Bin Oh, Kun Cho

**Affiliations:** 1 Biopharmaceutical Mass Spectrometry Research Unit, Korea Basic Science Institute, Cheongju 28119, Republic of Korea; rhfvm2@kbsi.re.kr (H.-J.Y.); kminsun@kbsi.re.kr (M.K.); 2 Department of Chemistry, Sogang University, Seoul 04100, Republic of Korea; 3 Division of Materials Science, Korea Basic Science Institute, Daejeon 34133, Republic of Korea; yhkim@kbsi.re.kr; 4 ASTA Corporation, 145 Gwanggyo-ro, Yeongtong-gu, Suwon 16229, Republic of Korea; woojin.jeon@astams.com (W.J.J.); byunggwun.jin@astams.com (B.G.J.); jyoh@astams.com (J.O.); hskim@astams.com (H.S.K.); yangskim@astams.com (Y.K.); 5 Department of Chemistry, Dongguk University, Seoul 04620, Republic of Korea; kimyk@dongguk.edu; 6 Department of Bio-Analytical Science, University of Science and Technology, Daejeon 34113, Republic of Korea

**Keywords:** lithium-ion battery anode, synthetic graphite, pitch-derived carbon coating, oxidation onset temperature, thermogravimetry–mass spectrometry, residual oxygen, active surface area

## Abstract

Graphite anodes are specified by bulk structural and textural parameters, but these converge after graphitization and say little about the surface at which the first cycle is determined. This work aimed to determine whether thermogravimetry coupled with time-of-flight mass spectrometry resolves precursor- and coating-specific surface behavior in graphite anodes and separates that behavior from artifacts of the measurement atmosphere. We examined nine anode materials—natural graphite, coal-tar-, and petroleum-derived artificial graphites, including an uncoated/coated commercial pair from each precursor and a carbonized/graphitized pair—using thermogravimetry coupled to time-of-flight mass spectrometry under helium, Raman spectroscopy, N_2_ physisorption, and electron microscopy. The CO_2_ evolved above 600 °C arose from oxidation by residual oxygen: O_2_ was consumed by 67–80%, while N_2_, Ar, and He changed by less than 13%, and the CO_2_ formed matched the available oxygen (*r* = 0.9988). We therefore did not compare CO_2_ yields and mass losses between runs, and we corrected oxidation onsets to a common oxygen level. Graphitization raised the onset by 225 °C at matched oxygen; coal-tar-derived graphites oxidized 6–27 °C later than petroleum-derived ones in all three precursor-matched comparisons; coating lowered the onset by 14–19 °C while moving the BET area in opposite directions, by −18% and +581%. An explicit sensitivity analysis shows that the first two results are independent of the correction, whereas the magnitude of the coating effect depends on it.

## 1. Introduction

Graphite remains the dominant negative-electrode material in commercial lithium-ion batteries (LIBs) and is expected to retain that position for the foreseeable future [1,2,3]. It is supplied in two forms. Natural graphite is mined and purified by acid leaching or high-temperature halogen treatment, then spheroidized and surface-coated before use [4]. Artificial graphite is manufactured by high-temperature treatment of carbonaceous feedstocks, principally coal-tar (CT) pitch and petroleum-derived residues such as pyrolysis fuel oil (PFO): the feedstock is coked and calcined, and the resulting carbon is graphitized above 2800 °C, industrially either in Acheson resistance furnaces, in which the packed charge itself forms the resistor, or in lengthwise-graphitization furnaces of the Castner type, in which the current passes along a column of shaped bodies [5,6]. Graphitic carbon can also be deposited directly from a hydrocarbon gas by chemical vapor deposition, a route used for coatings and for pyrolytic graphite but not at anode scale [7]. The two precursor families are chemically distinct at the outset: CT is strongly aromatic, contains quinoline-insoluble matter, and carries nitrogen and sulfur, whereas PFO is comparatively aliphatic and essentially free of heteroatoms [8,9]. These differences govern mesophase development during coking and therefore determine whether the resulting coke is optically isotropic or anisotropic [10,11]. However, after graphitization at temperatures approaching 3000 °C, the precursor-derived non-carbon elements volatilize. Sulfur and nitrogen are released between about 1500 and 2200 °C, hydrogen and oxygen are lost earlier during carbonization, and the inorganic residue—principally silicon, iron, aluminum, calcium, titanium, vanadium and nickel—leaves as the element, the carbide or, where a halogen is present, the volatile halide, so that total impurity contents fall to a few hundred parts per million or less [12]. Structural defects are healed at the same time, and the crystallographic signatures of the original precursor largely disappear [13,14].

Three problems follow from this manufacturing route and remain unresolved. First, energy and cost: graphitization dominates both the price and cradle-to-gate carbon footprint of artificial graphite, which is why researchers are actively pursuing electrochemical and catalytic routes operating far below 2800 °C [15]. The second is feedstock variability. Coal-tar and petroleum pitches are by-products whose composition is fixed by the parent refining or coking process rather than by the anode manufacturer, and the literature on them is dominated by studies of mesophase development and of the texture of the resulting coke [9,10,11] rather than by studies of the finished anode surface. The third, and the one addressed here, is verification. Comparisons of natural and artificial graphite anodes are generally framed in terms of reversible capacity, rate capability and cycle life [6,16], and direct comparisons between artificial graphites of different precursors are rare; where structural characterization is reported, it is almost always X-ray diffraction and Raman spectroscopy, both of which report bulk crystalline order. We are not aware of a study that compares the reactivity of coal-tar- and petroleum-derived anode graphites under matched thermal history, and the coated grades that dominate the market are in practice specified by a single textural number, the BET surface area, whose relation to chemical reactivity has been questioned since the active-surface-area concept was introduced [17,18,19].

The consequence is a characterization problem. We have recently shown that X-ray diffraction, Raman spectroscopy, transmission electron microscopy, and even half-cell electrochemical performance cannot distinguish artificial graphites prepared from CT and PFO precursors, whereas laser desorption/ionization mass spectrometry combined with multivariate statistics cleanly separates them [8]. The parameters conventionally used to characterize battery-grade graphite report bulk crystalline order that converges across processing routes.

The two graphite types also differ in how lithium enters them. Intercalation occurs through the prismatic edge planes; the basal planes are essentially impermeable unless they contain defects, so the accessible edge area and the length of the diffusion path within a particle together determine the rate at which lithium can be inserted and removed [16,20]. Reported solid-state diffusion coefficients in graphite span several orders of magnitude because they depend on the stage of intercalation, the measurement method, and whether the value refers to the crystallite or the whole particle [20]. Natural graphite is highly crystalline and strongly anisotropic, so flake particles must be spheroidized to shorten and randomize the diffusion path. Spheroidization exposes fresh edge surfaces, which are then coated. Artificial graphite is assembled from smaller crystallites in a more isotropic arrangement, shortening the internal path and improving rate capability at the cost of lower volumetric capacity [6,16]. In both cases, the prismatic edge sites that admit lithium are the same sites where the electrolyte is reduced on the first charge, so intercalation and irreversible loss cannot be optimized independently [20].

Interfacial behavior is governed by a different quantity. While the graphitic interior sets reversible capacity [21], the surface sets the first cycle. Oxygen-containing functional groups—carboxylic acids, lactones, anhydrides, phenols, carbonyls and quinones—reside at prismatic edge sites and lattice defects rather than on basal planes [22,23]. These sites initiate electrolyte reduction, nucleate the solid–electrolyte interphase, and irreversibly consume lithium inventory, so their population directly reflects the initial Coulombic efficiency [1,24,25,26]. Pitch-derived amorphous carbon coating is the standard industrial mitigation, intended to cover reactive edge sites with a more uniform and less reactive surface [1,27]. The same edge and defect sites also govern carbon reactivity toward oxygen, so oxidation behavior is a sensitive probe of the reactive surface [17,18].

In practice, however, coated grades are specified largely by BET surface area. This is a questionable proxy. Nitrogen physisorption yields the total surface area, which, in graphite, is dominated by basal planes; the chemically reactive fraction, the active surface area associated with edge and defect sites, is a small and variable subset of it [17,18,19]. A coating that fills surface asperities and one that deposits a microporous amorphous layer can shift the BET area in opposite directions while producing the same chemical result, and no area measurement alone can distinguish the two cases.

Evolved-gas analysis offers a route to the reactive surface that bulk parameters miss. Heating carbon while monitoring evolved gases by mass spectrometry [28] probes the population and reactivity of edge and defect sites, either through thermal decomposition of surface oxygen complexes under inert flow [29,30,31] or through the onset of oxidation when oxygen is present [32,33]. The two regimes are easily confused. Hyphenated thermogravimetry–mass spectrometry systems are not perfectly leak-tight, and residual oxygen in a nominally inert atmosphere is known to burn graphite at high temperature, producing CO_2_ while the O_2_ signal falls [34]. For well-graphitized carbon, which has a small inventory of surface oxygen groups, such a trace can dominate the CO_2_ signal. Distinguishing the two requires tracking the oxygen signal itself, which is straightforward with a time-of-flight analyzer that continuously records the full mass spectrum but is easily missed when monitoring only selected ions.

This work aims to determine whether evolved-gas analysis by thermogravimetry coupled to time-of-flight mass spectrometry resolves precursor- and coating-specific differences between graphite anode materials that bulk structural and textural parameters do not resolve. Four objectives follow from it: (i) to establish the origin of the CO_2_ evolved from graphite above 600 °C under nominally inert gas, and thereby to determine which measured quantities are properties of the material and which are properties of the measurement atmosphere; (ii) to quantify the effect of a pitch-derived carbon coating on oxidation onset, Raman disorder and texture within matched uncoated/coated pairs of the same commercial base material; (iii) to test whether coal-tar- and petroleum-derived graphites differ in oxidation onset when compared at matched thermal history, and whether any such difference is visible in Raman parameters or BET area; and (iv) to determine how far these conclusions depend on the correction applied for residual oxygen.

We selected nine graphite anode materials to meet these objectives: natural graphite; commercial coal-tar- and petroleum-derived artificial graphites, each in uncoated and coated forms; in-house isotropic and anisotropic coal-tar-derived graphites and an anisotropic petroleum-derived graphite; and petroleum-derived anisotropic coke examined after carbonization at 1350 °C and after graphitization at 3000 °C. We measured all samples by TGA–TOF MS under helium and characterized them by Raman spectroscopy, nitrogen physisorption, and scanning electron microscopy.

## 2. Materials and Methods

### 2.1. Materials

We examined nine carbon anode materials (Table 1). Five of them—#01, #03, #05, #07 and #09—are commercial anode grades obtained from Korean anode manufacturers under confidentiality agreements that do not permit the suppliers or their grade designations to be named; the highest heat-treatment temperature applied by those suppliers is likewise not disclosed to us. Sample #01 was a commercial coated natural graphite. Samples #02, #04, #06 and #08 were prepared in-house from refined coal tar (CT; POSCO, Pohang, Republic of Korea) and pyrolysis fuel oil (PFO; LG Chem, Seoul, Republic of Korea) pitches following the route described previously [8]: the refined soft pitches were coked at 480 °C for 7 h under 3.5 bar of nitrogen, carbonized at 1350 °C under atmospheric-pressure nitrogen to give calcined cokes of particle size below 74 μm, and graphitized at 3000 °C in an induction furnace. Sample #06 is the petroleum-derived anisotropic carbonized product, retained deliberately at the pre-graphitization stage, and sample #08 is the same material after graphitization. Samples #03 and #09 were commercial CT- and PFO-derived graphite anodes, and samples #05 and #07 were the pitch-coated grades of those same two commercial base materials.

The set therefore contains three matched pairs that isolate a single process variable: #03/#05 and #07/#09 differ only in the presence of the carbon coating, and #06/#08 differ only in whether the 3000 °C graphitization step was applied. All powders were used as received; we performed no drying or pre-treatment before thermal analysis. Each sample was measured once under the program described in Section 2.2. Sample #01 was also measured on a second day at a different residual oxygen level to calibrate the oxygen correction described in Section 2.3.

### 2.2. Thermogravimetric Analysis Coupled to Time-of-Flight Mass Spectrometry

Evolved-gas analysis was performed on a TGA–TOF MS comprising a Discovery TGA 550 thermogravimetric analyzer (TA Instruments, New Castle, DE, USA) and a time-of-flight mass spectrometer (GM-TOF for TGA; ASTA Corporation, Suwon, Republic of Korea), hyphenated through a heated gas transfer line that prevents condensation of the evolved species [28]. We performed measurements in the thermogravimetric analyzer’s evolved-gas analysis (EGA) furnace using an open, high-temperature platinum pan.

Between 39.45 and 72.72 mg of powder (Table 2; run records in Table S1) was held at the starting temperature under flowing helium (99.999%, 70 mL min^−1^) for approximately 21 min (13 min for #04). It was then heated to 1000 °C at a nominal 20 °C min^−1^, the rate measured from the recorded temperature record being 20.17 °C min^−1^, and held at 1000 °C for a further 10 min; this final segment is required for the sample to reach the set point, and the sample temperature exceeded 999 °C for the last 7.2 min of it. Temperature was calibrated against the Curie point of nickel, most recently on 5 February 2025. The transfer line was held at 250 °C throughout. Evolved species were ionized by electron impact at 80 eV, the setting found to optimize sensitivity and resolution for this analyzer (library reference spectra being recorded at 70 eV), and mass spectra were acquired over *m*/*z* 1–300. The thermobalance recorded sample mass at 10 Hz. The time-of-flight analyzer was operated at a pulse repetition rate of 8 kHz, and spectra were summed over 10 s intervals, corresponding to 3.3 °C between successive recorded spectra during the ramp; this interval sets the intrinsic resolution of the gas-evolution onset temperatures reported here, and is small relative to the differences discussed in Section 3.4, Section 3.5 and Section 3.6.

All analyses used the full-range spectra (*m*/*z* 1–300) as recorded; we did not use the smoothed channel subsets provided in the instrument’s routine export because the smoothing delays the signals relative to temperature by up to about 20 °C. We extracted ion currents at *m*/*z* 4 (He), 18 (H_2_O), 28 (N_2_, CO), 32 (O_2_), 40 (Ar), and 44 (CO_2_), and surveyed the remaining channels for other evolved species. For the #01 replicate, whose thermogravimetric record was not retained, we placed the spectra on the temperature axis using the common temperature program and aligned them to the end of the run; across the other nine runs, this alignment reproduces the recorded temperature to within 1.3 °C (standard deviation) over 500–750 °C.

### 2.3. Residual Oxygen, Onset Temperatures and Oxygen Correction

We characterized the residual oxygen level of each run by the mean *m*/*z* 32 ion current over 100–300 °C, a range in which no CO_2_ is evolved, and by oxygen consumption, measured as the fractional decrease in *m*/*z* 32 between that baseline and 900–999 °C. We made the same comparison for *m*/*z* 4, 28, and 40, which serve as inert references for changes in flow or ion-source response.

Onset temperatures were determined using a single explicit criterion applied identically to both signals and all samples. Each signal was restricted to the heating ramp, smoothed with a seven-point, second-order Savitzky–Golay filter, and referenced to its mean over 100–300 °C; the excursion from this baseline was taken as the rise in *m*/*z* 44 ion current for CO_2_ and as the mass lost for the thermogravimetric curve. We defined the onset as the temperature at which the excursion first reaches, and thereafter remains above, 10% of its maximum during the ramp, determined by linear interpolation. The 10% criterion is the fractional-height equivalent of the conventional tangent-intersection construction—for a sigmoidal transition, the tangent at the inflection point meets the baseline at 10.5% of the step height for an error-function profile and 11.9% for a logistic one—but, unlike the tangent construction, it does not depend on analyst-selected limits.

Because the rate of carbon oxidation depends on the oxygen partial pressure to an order less than unity [32,33], a fixed-fraction onset shifts to lower temperatures when less oxygen is available. The sensitivity was calibrated from two runs of sample #01 at residual *m*/*z* 32 levels of 36.4 × 10^3^ and 13.6 × 10^3^ counts, yielding s = Δ*T*/ln(*P*_1_/*P*_2_) = 25.6 °C per *e*-fold change in residual oxygen. Onsets were corrected to the median residual oxygen level of the nine samples, Pref = 47.6 × 10^3^ counts, using

*T*_corr_ = *T* + s ln(*P*_ref_/*P*). (1)

A sensitivity of this form follows from the rate law: if the oxidation rate varies as *P*(O_2_)*^n^* exp(−*E*_a_/RT), then a fixed-fraction onset shifts with oxygen pressure as d*T*/dln*P* = −*nRT*^2^/*E*_a_. With the activation energy of 179 kJ mol^−1^ reported for graphite oxidation [33] and the mean onset temperature of the two #01 runs (929 K), the measured sensitivity corresponds to an apparent reaction order in O_2_ of 0.64, below unity as expected for the carbon–oxygen reaction in the chemically controlled regime [32,33]. The dependence of the reported comparisons on the value of s is examined explicitly in Section 3.3. We repeated the procedure at 5% and 20% thresholds, corresponding to s = 15.9 and 29.7 °C, respectively.

CO_2_ yields, obtained by integrating the baseline-subtracted *m*/*z* 44 current against temperature over 500–1000 °C and normalizing to initial sample mass, and total mass losses are reported for completeness but are not compared between samples, because both scale with the oxygen supplied (Section 3.3). Because oxidation and desorption temperatures depend on heating rate [35], the absolute onsets reported here are not directly comparable with temperature-programmed studies conducted at other heating rates; all runs used the same program, so comparisons between them are unaffected.

### 2.4. Raman Spectroscopy

We recorded Raman spectra at the Korea Basic Science Institute (KBSI) on a RAMANtouch microspectrometer (Nanophoton Corp., Osaka, Japan) using 532.04 nm excitation at 1 mW, a 600 groove mm^−1^ grating, a 50 μm slit, and an LU Plan Fluor ×100 objective (NA 0.9). We accumulated each spectrum for 10 s over three exposures and measured five independent positions per sample. Spectra were baseline-corrected with a linear function over 1100–1750 cm^−1^ and 2550–2850 cm^−1^, and the D, G, and 2D bands were fitted with Lorentzian profiles by non-linear least squares (SciPy 1.17, Python 3.12) and interpreted following [36,37,38]; individual fit parameters are given in Table S2. We report peak intensity ratios, band positions, and full widths at half maximum as the mean ± standard deviation from five measurements.

### 2.5. Nitrogen Physisorption

Surface area and pore structure were determined on an ASAP 2420 analyzer (V2.09; Micromeritics Instrument Corp., Norcross, GA, USA) at the Center for Research Facilities, Chungnam National University. Between 0.86 and 2.10 g of each powder was degassed under vacuum at 25 °C for 4 h. Degassing was carried out at ambient temperature rather than at the 200–300 °C conventionally applied to carbon adsorbents because heating in that range initiates decomposition of the surface groups that are the subject of this study, so that the textural and evolved-gas data refer to the same as-received surface state. Nitrogen isotherms were measured at 77.3 K with an equilibration interval of 10 s and no thermal correction. All samples exhibited type II isotherms according to the IUPAC classification [39]. BET areas were calculated over P/P_0_ = 0.057–0.180 using a nitrogen cross-sectional area of 0.1620 nm^2^; correlation coefficients exceeded 0.9999, and *C* constants ranged from 77 to 429 (Table S3). External area and micropore volume were obtained by the t-plot method with the Harkins–Jura thickness equation over a statistical thickness of 0.35–0.50 nm. Total pore volume was the single-point adsorption value at P/P_0_ ≈ 0.99, and pore size distributions were calculated by the BJH method with the Faas correction over 1.7–300 nm.

### 2.6. Scanning Electron Microscopy

Particle morphology was examined at the Kumho Petrochemical Central Research Institute (Daejeon, Republic of Korea) on a JSM-7500F cold field-emission scanning electron microscope (JEOL Ltd., Tokyo, Japan) using secondary electron imaging [40] at an accelerating voltage of 20 kV and a working distance of 6.0 mm. We mounted powders on conductive carbon tape and imaged them at ×1000 in low-magnification mode; we did not apply a conductive coating, as the materials were sufficiently conductive as received.

### 2.7. Data Analysis

We computed onset temperatures, correlation coefficients, and group statistics in Python 3.12 using NumPy 2.1 and SciPy 1.17. We report both Pearson and Spearman coefficients; a Pearson coefficient that is not reproduced by the corresponding Spearman coefficient, or that does not survive removal of an extreme point, is treated as leverage-driven. Group comparisons use Welch’s *t*-test. For groups of three and five, or two and three, samples, the Mann–Whitney U test cannot reach conventional significance even under complete separation, so we prioritize comparisons between samples sharing a base material or a synthesis route over group-level tests. Generative artificial intelligence was used as a computational and writing aid in this work: it assisted in writing the Python routines used for onset determination, for the residual-oxygen correction of Equation (1), for the Lorentzian fitting of the Raman bands and for the statistical analysis; in producing exploratory versions of the figures, which were subsequently redrawn by the authors in OriginPro 2025b from the same tabulated values; and in preparing an initial English draft from the authors’ outline, data tables and written interpretation. The authors verified all numerical results against the raw instrument files, and they additionally confirmed the onset temperatures by manually constructing the tangent intersections. We designed the experiment, performed the measurements, interpreted the results, and drew the conclusions.

## 3. Results

### 3.1. Particle Morphology

**Figure 1:**
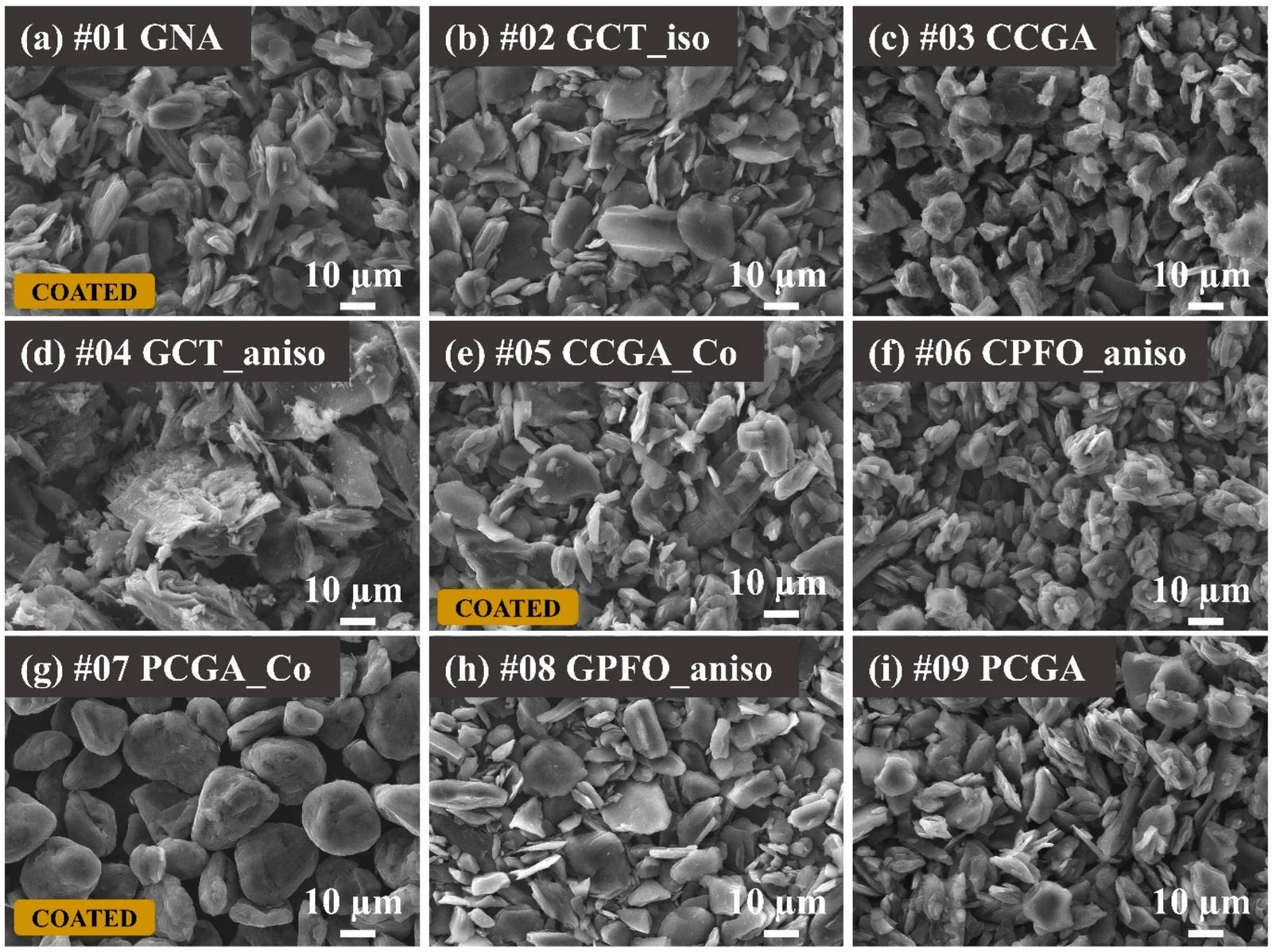
Secondary electron micrographs of the nine carbon anode materials at ×1000 magnification, arranged by precursor: (**a**) #01 GNA, natural, coated; (**b**) #02 GCT_iso; (**c**) #03 CCGA; (**d**) #04 GCT_aniso; (**e**) #05 CCGA_Co, the coated form of (**c**); (**f**) #06 CPFO_aniso, carbonized at 1350 °C; (**g**) #07 PCGA_Co, the coated form of (**i**); (**h**) #08 GPFO_aniso, the graphitized form of (**f**); (**i**) #09 PCGA. Coating smooths the surfaces of both commercial grades and rounds the petroleum-derived particles, but leaves sharp crystal faces on the natural graphite in (**a**). JSM-7500F cold field-emission microscope, secondary electron imaging at 20 kV, working distance 6.0 mm, low-magnification mode; scale bars 10 μm.

Secondary electron imaging at ×1000 (Figure 1) resolves the sample set along two axes. The anisotropic in-house materials #04, #06, and #08 exhibit lamellar, plate-like particles with a well-developed layered structure, whereas the isotropic materials are more equant and irregular in habit. Superimposed on this is the effect of coating, which is directly visible in both matched pairs: the uncoated commercial graphites #03 and #09 show pronounced surface undulation with exposed layer edges, while their coated counterparts #05 and #07 present smoother exteriors, with #07 additionally rounded to predominantly spheroidal particles. Sample #06, carbonized but not graphitized, is distinguished by a mixed acicular and platy habit with densely aggregated fine particles.

The coated natural graphite #01 is the exception. Its particles retain distinct crystal faces and sharply defined layer edges and show no surface smoothing, unlike #05 and #07, indicating incomplete or heterogeneous coating coverage rather than the continuous film present on the two commercial artificial graphites. The Raman measurements (Section 3.5 and Section 3.7) reach the same conclusion independently, with #01 alone showing a standard deviation exceeding its mean.

Morphology is reported here qualitatively and is not used to support any quantitative claim. It establishes that the surface modification inferred from spectroscopy and evolved-gas analysis is directly observable, and it documents the one sample in which that modification is incomplete.

### 3.2. High-Temperature CO_2_ Arises from Oxidation by Residual Oxygen

A survey of the full-range spectra showed that only *m*/*z* 44, its neighboring and fragment channels (*m*/*z* 12, 43, and 45), and *m*/*z* 18 increased by more than 10 times their baseline noise between 300 and 1000 °C in any sample. No sulfur- or nitrogen-bearing species (SO_2_, SO, H_2_S, COS, CS_2_, HCN, NO, NO_2_), hydrocarbons, or H_2_ were detected; no CO was resolved above the nitrogen background at *m*/*z* 28.

**Figure 2:**
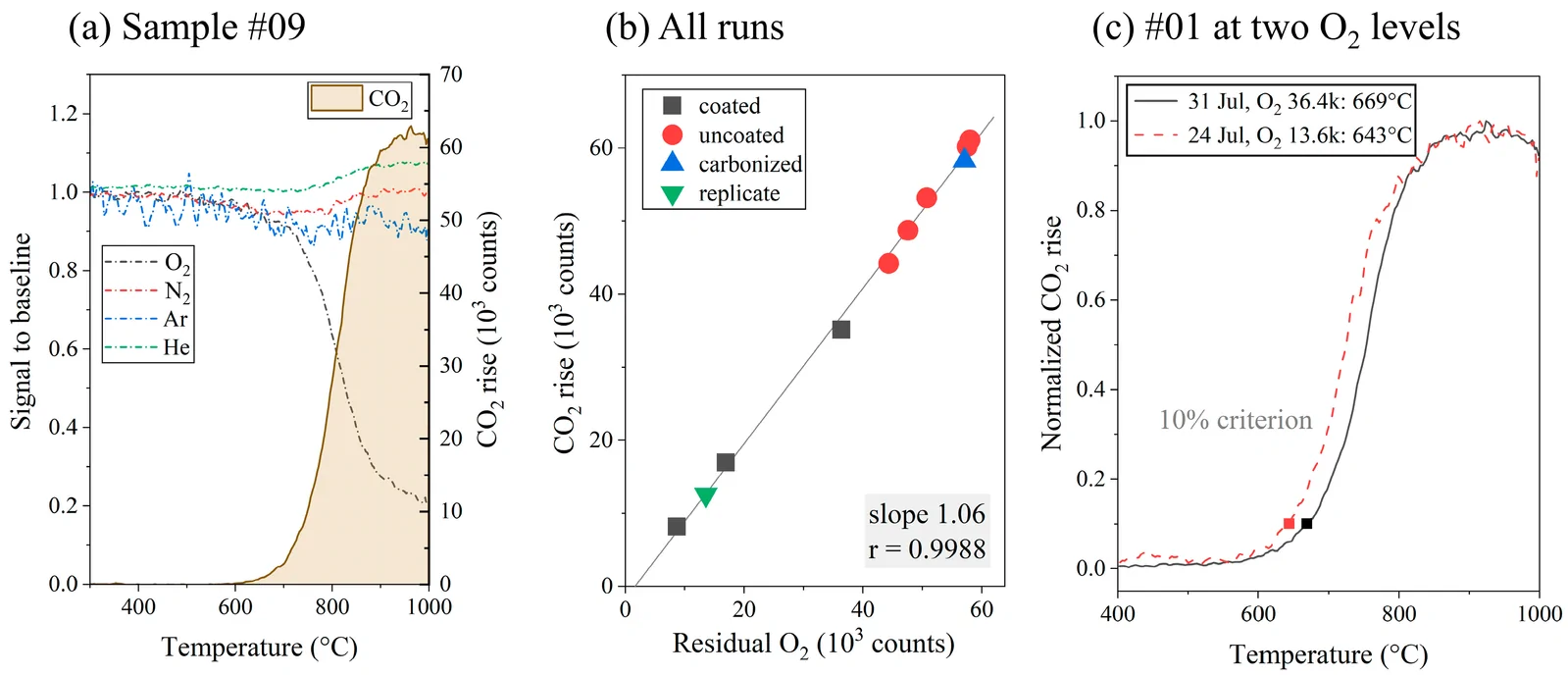
Identification of residual oxygen as the source of high-temperature CO_2_. (**a**) Sample #09: ion currents for O_2_ (*m*/*z* 32), N_2_ (*m*/*z* 28), Ar (*m*/*z* 40), and He (*m*/*z* 4) relative to their 100–300 °C baselines, and the rise in CO_2_ (*m*/*z* 44; right axis); O_2_ is consumed as CO_2_ forms while the inert species remain constant. (**b**) CO_2_ rise at 900–999 °C against residual O_2_ for all ten runs, including the #01 replicate; line: least-squares fit (slope 1.06, *r* = 0.9988). (**c**) Normalized CO_2_ rise for the two runs of #01 at residual O_2_ levels of 36.4 × 10^3^ and 13.6 × 10^3^ counts; the lower oxygen level displaces the curve and the 10% onset to a lower temperature by 25 °C.

The *m*/*z* 32 signal, however, fell steeply as CO_2_ evolved (Figure 2a). Between the 100–300 °C baseline and 900–999 °C, O_2_ decreased by 67–80% (mean 75%) in all ten runs, whereas He, N_2_, and Ar changed by less than 13%, excluding a change in flow or ion-source response. The decrease in O_2_ tracked the rise in CO_2_ through the transition, and the CO_2_ formed above 900 °C was proportional to the residual oxygen available (Figure 2b; slope 1.06, r = 0.9988, *n* = 10), with a constant ratio of CO_2_ formed to O_2_ consumed of 1.34 ± 0.07 in ion-current units. Extrapolated to zero residual oxygen, the CO_2_ increase is −4.3% of its mean value, with an upper 95% bound of −0.4%, so no oxygen-independent CO_2_ is detectable. Therefore, the CO_2_ evolved at high temperature results from carbon oxidation by residual oxygen, not from thermal decomposition of surface oxygen groups.

The residual oxygen level varied sevenfold between runs, from 8.7 × 10^3^ to 58.0 × 10^3^ counts (Table 2; run dates in Table S1), and on each measurement day it increased with successive runs; on 25 July it rose from 16.9 × 10^3^ to 57.1 × 10^3^ over four consecutive samples. The runs on the coated grades #05 and #07 were the first of their respective days and had residual oxygen levels of 38% and 15% of those of their uncoated counterparts.

### 3.3. Which Quantities Can Be Compared

**Figure 3:**
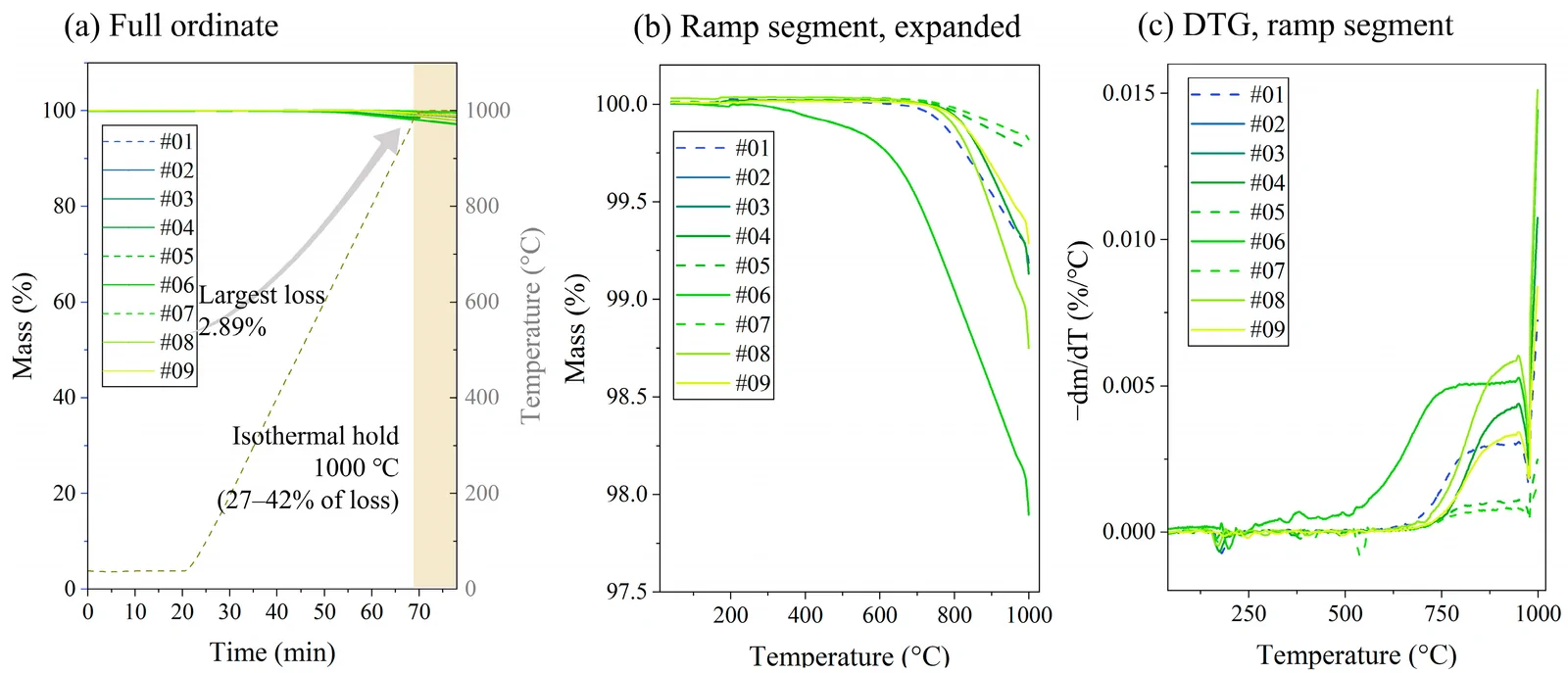
Thermogravimetric behavior of the nine materials under flowing helium (70 mL min^−1^): (**a**) mass retention against time over the complete program on the full 0–100% ordinate, with the temperature program (dotted line, right axis) and the terminal isothermal hold shaded; (**b**) the ramp segment expanded over 97.5–100.2%; (**c**) derivative thermogravimetric curves for the ramp segment. The mass loss reflects oxidation of the carbon by residual oxygen (Section 3.2) and differs between runs mainly because the oxygen level differed. Coated samples are drawn with dashed lines.

Total mass loss (0.27–2.89%; Figure 3, Table 2) and the integrated CO_2_ yield correlate with the residual oxygen level (*r* = 0.80 and 0.77; 0.89 for both when #06 is excluded) and with one another (*r* = 0.994, Figure S1), as expected if both record the carbon burnt by the oxygen supplied. Neither is therefore a property of the material alone, and neither is compared between samples below.

**Figure 4:**
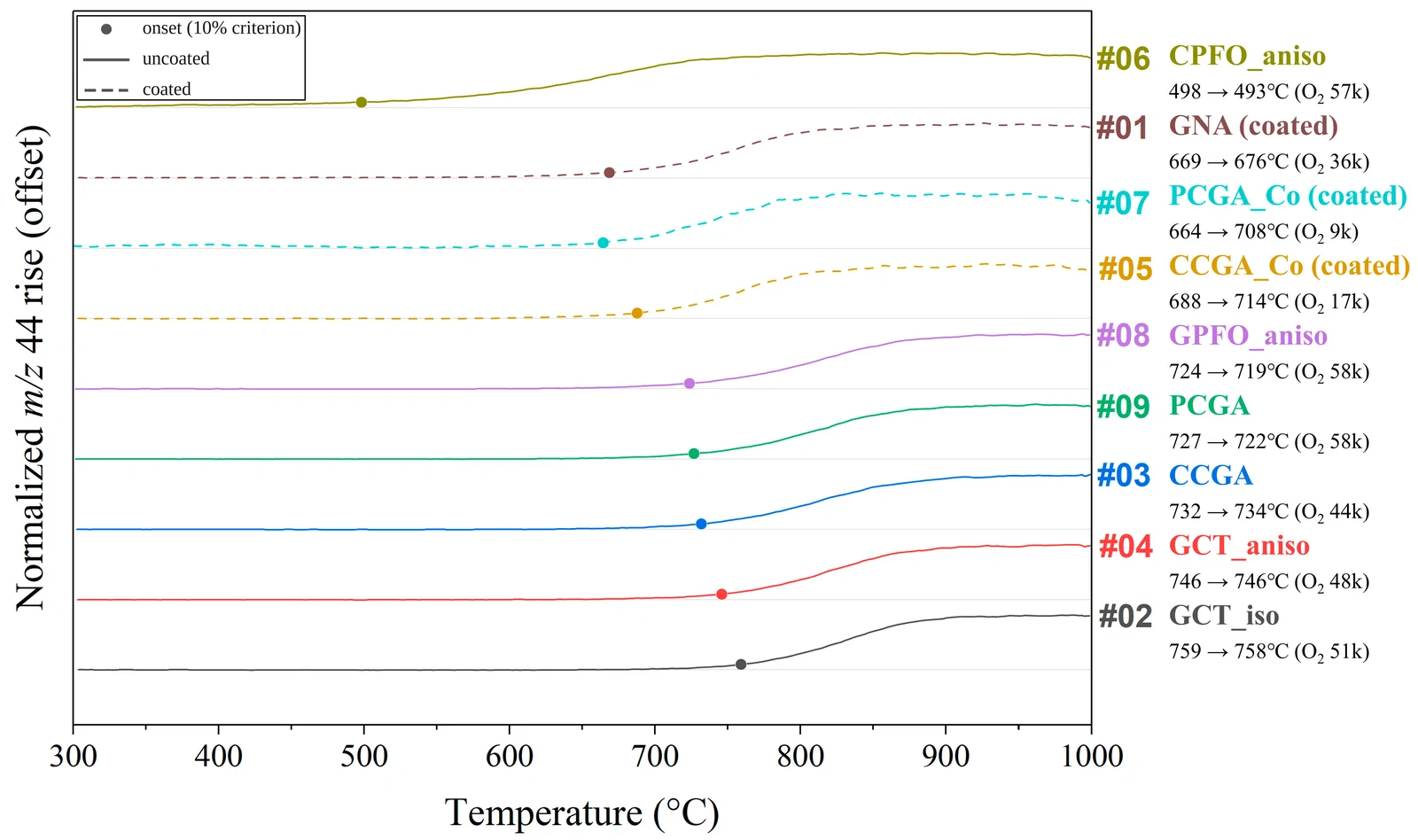
Normalized rise in *m*/*z* 44 ion current against temperature for the nine materials, offset vertically and ordered by corrected oxidation onset. Markers show the as-measured onset at 10% of the maximum rise; labels give the as-measured and O_2_-corrected onsets and the residual O_2_ level of each run. Coated samples are drawn with dashed lines.

Onset temperatures are normalized to each transition’s size and are therefore less directly affected, but they still shift with residual oxygen. The two runs of sample #01 gave CO_2_ onsets of 668.7 °C and 643.5 °C at residual oxygen levels of 36.4 × 10^3^ and 13.6 × 10^3^ counts (Figure 2c): a 2.7-fold reduction in oxygen lowered the onset by 25 °C, and by 16 °C and 29 °C at the 5% and 20% thresholds. The corrected onsets in Table 2 and Figure 4 remove this dependence to first order. The correction is small (less than 7 °C) for all uncoated samples, whose residual oxygen levels are within 30% of the reference, but it raises the onsets of the coated grades #05 and #07 by 26 °C and 44 °C, respectively.

In all 9 samples, the CO_2_ onset preceded the mass-loss onset by 75 ± 19 °C (range 57–112 °C), so the mass spectrometer detected oxidation well before any change in mass could be registered by the thermobalance.

Because the correction rests on a single replicate pair, the dependence of each comparison on the assumed value of s was examined explicitly rather than assumed away. For two runs made at residual oxygen levels *P_i_* and *P_j_*, the corrected difference varies linearly with *s*,

Δ*T*_corr_ = Δ*T*_meas_ + s ln(*P*_j_/*P*_i_), (2)

so the value of s at which any comparison would change sign can be stated exactly (Table 3). The graphitization comparison is independent of s, because the two runs were made at the same oxygen level. Two of the three precursor comparisons preserve their direction for all non-negative s, and the third does so for s < 35 °C per e-fold, 1.4 times the calibrated value. The coating comparisons are the most sensitive: the direction is preserved for s < 46 °C per e-fold in the coal-tar pair and s < 33 °C per e-fold in the petroleum pair, up to 1.8 and 1.3 times the calibrated value, respectively. The magnitude of the coating effect therefore depends on the correction model, whereas the precursor and heat-treatment results do not; we carry this distinction through the Discussion and restate it in Section 4.6.

**Table 3:** Dependence of the principal comparisons on the oxygen-correction coefficient s. Each entry is the difference in oxidation onset between the two runs named, evaluated from Equation (2) at the value of s in the column heading; the first sample listed in each comparison is *i* and the second is *j*. The column s = 25.6 °C per e-fold is the value calibrated from the #01 replicate pair and used throughout, and reproduces the O_2_-corrected differences of Table 4 and Table 5; s = 0 is the difference as measured, with no correction applied, and 12.8, 38.4 and 51.2 °C per e-fold are 0.5, 1.5 and 2.0 times the calibrated value. Because each difference is linear in s, the value at which it would change sign is exact.

Comparison	*P*_i_/*P*_j_ (10^3^ Counts) ^a^	ln(*P*_j_/*P*_i_)	Onset difference (°C) at *s* = ^b^					Sign Reverses at ^c^
			0	12.8	25.6	38.4	51.2	
Graphitization (#08 − #06) ^d^	57.5/57.1	−0.0072	+225.6	+225.5	+225.4	+225.3	+225.2	None ^e^
Precursor, in-house (#04 − #08)	47.6/57.5	+0.1901	+22.2	+24.6	+27.1	+29.5	+31.9	None for s ≥ 0
Precursor, uncoated (#03 − #09)	44.3/58.0	+0.2692	+5.1	+8.5	+12.0	+15.4	+18.9	None for s ≥ 0
Precursor, coated (#05 − #07)	16.9/8.7	−0.6660	+23.4	+14.9	+6.4	−2.1	−10.7	35 °C (1.4 × s)
Coating, coal tar (#05 − #03)	16.9/44.3	+0.9630	−44.0	−31.7	−19.4	−7.1	+5.3	46 °C (1.8 × s)
Coating, petroleum (#07 − #09)	8.7/58.0	+1.8982	−62.4	−38.1	−13.8	+10.5	+34.8	33 °C (1.3 × s)

^a^ Mean *m*/*z* 32 ion current over 100–300 °C, from Table 2. ^b^ In °C per e-fold change in residual oxygen. ^c^ Value of s at which the sign of the difference would reverse, with that value as a multiple of the calibrated 25.6 °C per e-fold; given to the nearest degree, since s is itself calibrated from one replicate pair. ^d^ Carbonized coke #06 against the same coke after graphitization at 3000 °C(#08). ^e^ The two runs were made at effectively the same residual oxygen level, so this comparison carries no practical dependence on s; the formal crossing point exceeds 3 × 10^4^ °C per e-fold.

**Table 4:** Matched-pair comparisons. Ratios and differences are after/before.

Pair	Variable	Residual O_2_ Ratio	ΔOnset, as Measured	ΔOnset, O_2_-Corrected	I(D)/I(G)	ΔBET Area	H_2_O Released, 40–300 °C
#03 → #05	Coating (coal tar)	0.38	−44.0 °C	−19.4 °C	0.015 → 0.101	−18%	×0.95
#09 → #07	Coating (petroleum)	0.15	−62.4 °C	−13.8 °C	0.030 → 0.211	+581%	×126
#06 → #08	Graphitization 1350 → 3000 °C	1.01	+225.6 °C	+225.4 °C	0.836 → 0.036	−21%	×0.37

**Table 5:** Precursor-matched comparisons of oxidation onset (10% criterion).

Comparison	Coal Tar	Petroleum	Residual O_2_ Ratio (Petroleum/Coal Tar)	Δ as Measured (Coal Tar − Petroleum)	Δ O_2_-Corrected
In-house anisotropic, graphitized	#04 GCT_aniso	#08 GPFO_aniso	1.21	+22.2 °C	+27.1 °C
Commercial, uncoated	#03 CCGA	#09 PCGA	1.31	+5.1 °C	+12.0 °C
Commercial, coated	#05 CCGA_Co	#07 PCGA_Co	0.51	+23.4 °C	+6.4 °C

### 3.4. Carbon Coating: A Common Effect on Reactivity, Precursor-Specific Effects on Texture

**Figure 5:**
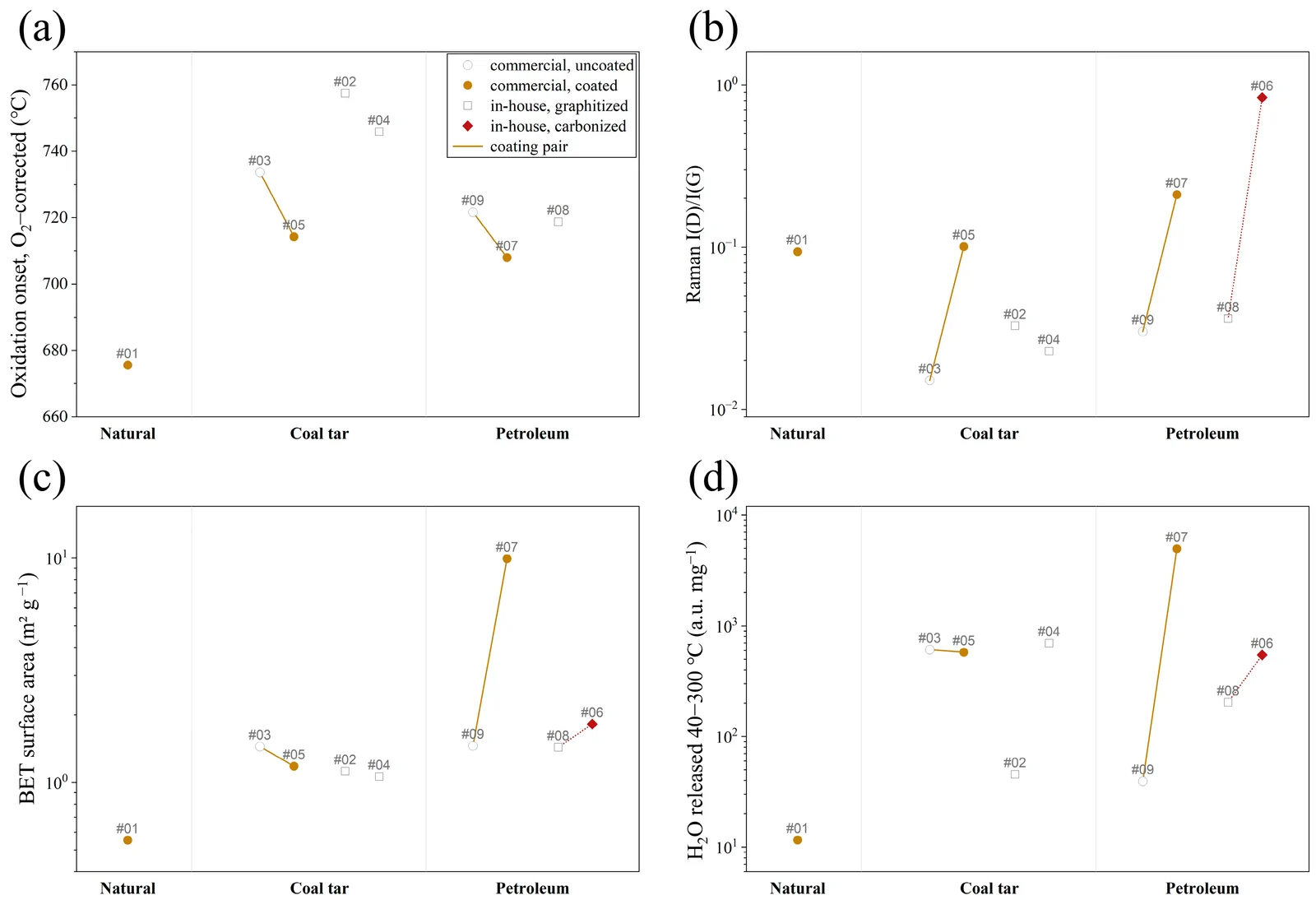
Precursor- and coating-specific behavior: (**a**) O_2_-corrected oxidation onset; (**b**) Raman I(D)/I(G) ratio; (**c**) BET surface area; (**d**) H_2_O released between 40 and 300 °C, normalized to sample mass. Samples are grouped by precursor; circles denote commercial and squares in-house graphitized materials, filled symbols coated materials, and the diamond the carbonized coke #06 (off scale in (**a**)). Solid lines connect the uncoated and coated forms of each commercial graphite; dotted lines connect #06 with its graphitized form #08. Coating shifts (**a**,**b**) in the same direction for both precursors, but shifts (**c**,**d**) in opposite directions.

Each artificial precursor is represented by commercial graphite in uncoated and coated forms, so we can assess the effect of coating within a single base material (Table 3, Figure 5). In both pairs, coating lowered the oxidation onset by 44 °C and 62 °C, as measured, and by 19 °C and 14 °C after correction for residual oxygen; the direction was the same at the 5%, 10%, and 20% thresholds. In both pairs, it also raised the Raman I(D)/I(G) ratio by a factor of about seven, from 0.015 to 0.101 for the coal-tar-derived grade and from 0.030 to 0.211 for the petroleum-derived grade, and broadened the D band from 28–33 cm^−1^ to 143–156 cm^−1^, the signature of a disordered carbon overlayer.

In texture, however, the two coatings are opposite. Coating the coal-tar-derived graphite lowered the BET area by 18%, left low-temperature water release essentially unchanged, and produced smoother particle surfaces (Section 3.1). Coating the petroleum-derived graphite increased the BET area by 581% (from 1.46 to 9.92 m^2^ g^−1^), and this increase is microporous in character. The t-plot external area of #07 is 6.96 m^2^ g^−1^, so 2.96 m^2^ g^−1^, or 30% of the BET area, is contributed by pores narrower than the t-plot range; #07 carries the only positive t-plot micropore volume in the set (0.00129 cm^3^ g^−1^, against values indistinguishable from zero for the other eight), the highest BET C constant (429, against 77–261), and the smallest mean pore width (8.4 nm, against 17.3 nm for its uncoated counterpart #09). Total pore volume rose by 3.3-fold, and water released below 300 °C increased by 126-fold (Figures S2 and S3, Table S3), and the particles became predominantly spheroidal. The coal-tar-derived coating thus fills and smooths the surface, whereas the petroleum-derived coating deposits a microporous, hydrophilic amorphous layer; both nonetheless shift reactivity and disorder in the same direction.

Across all 8 graphitized samples, the three coated materials remained below the five uncoated ones after correction, oxidizing at 676–714 °C versus 719–758 °C, but the gap narrowed from 36 °C to 4.5 °C, and the group difference was not statistically significant (Welch *t*-test, *p* = 0.07). The coating effect on oxidation onset is therefore consistent in direction but modest in magnitude, and its size depends on the oxygen correction.

### 3.5. Origin of the Precursor Difference

Three comparisons isolate precursor on otherwise similar materials: the in-house anisotropic graphites prepared by the same route from coal tar (#04) and pyrolysis fuel oil (#08), and the commercial coal-tar- and petroleum-derived graphites in uncoated (#03, #09) and coated (#05, #07) form. In all three, the coal-tar-derived material oxidized later (Table 5), by 27, 12, and 6 °C after correction and by 22, 5, and 23 °C as measured. The trend persisted in the coated comparison, although the petroleum-derived run had half the residual oxygen of the coal-tar-derived one, so this is not an artifact of oxygen level. Grouped together, the three uncoated coal-tar-derived graphites oxidized at 734–758 °C (corrected), and the two petroleum-derived ones at 719–722 °C, with no overlap at the 10% and 20% thresholds (Welch *t*-test, *p* = 0.06). By contrast, the Raman ratios overlap between the two precursor families (I(D)/I(G) = 0.015–0.033 and 0.030–0.036 for uncoated coal-tar- and petroleum-derived graphites), so the oxidation onset registers a precursor difference that the bulk structural parameter does not.

Among the coal-tar-derived in-house materials, isotropic graphite #02 oxidized 12 °C later than anisotropic #04, a smaller difference than those associated with precursor or coating. The coated natural graphite #01 oxidized earliest among the coated materials, at 676 °C, compared with 708–714 °C for the coated artificial grades. Its Raman parameters also set it apart: its five I(D)/I(G) measurements ranged from 0.007 to 0.293, an order of magnitude wider than any other sample, and its D band was 53 cm^−1^ wide, compared with 143–156 cm^−1^ for the other two coated grades. Together with the retained crystal faces seen by SEM (Section 3.1), these observations indicate a thin or discontinuous coating that leaves part of the natural graphite surface exposed.

### 3.6. Significance of Heat-Treatment History

Sample #06, the anisotropic petroleum-derived coke carbonized at 1350 °C and oxidized at 493 °C (182 °C below any graphitized sample), showed a graphitization onset at 3000 °C (#08), which was 225 °C higher (Table 4). Because the residual oxygen levels of the two runs were almost identical (57.1 × 10^3^ and 57.5 × 10^3^ counts), this comparison requires no correction and is the most robust result of the study; the increase was 279, 225, and 190 °C at the 5%, 10%, and 20% thresholds. Graphitization also reduced I(D)/I(G) from 0.836 to 0.036, the BET area by 21%, and the low-temperature water release by 63%.

### 3.7. Raman and BET Parameters as Predictors of Oxidation Onset

**Figure 6:**
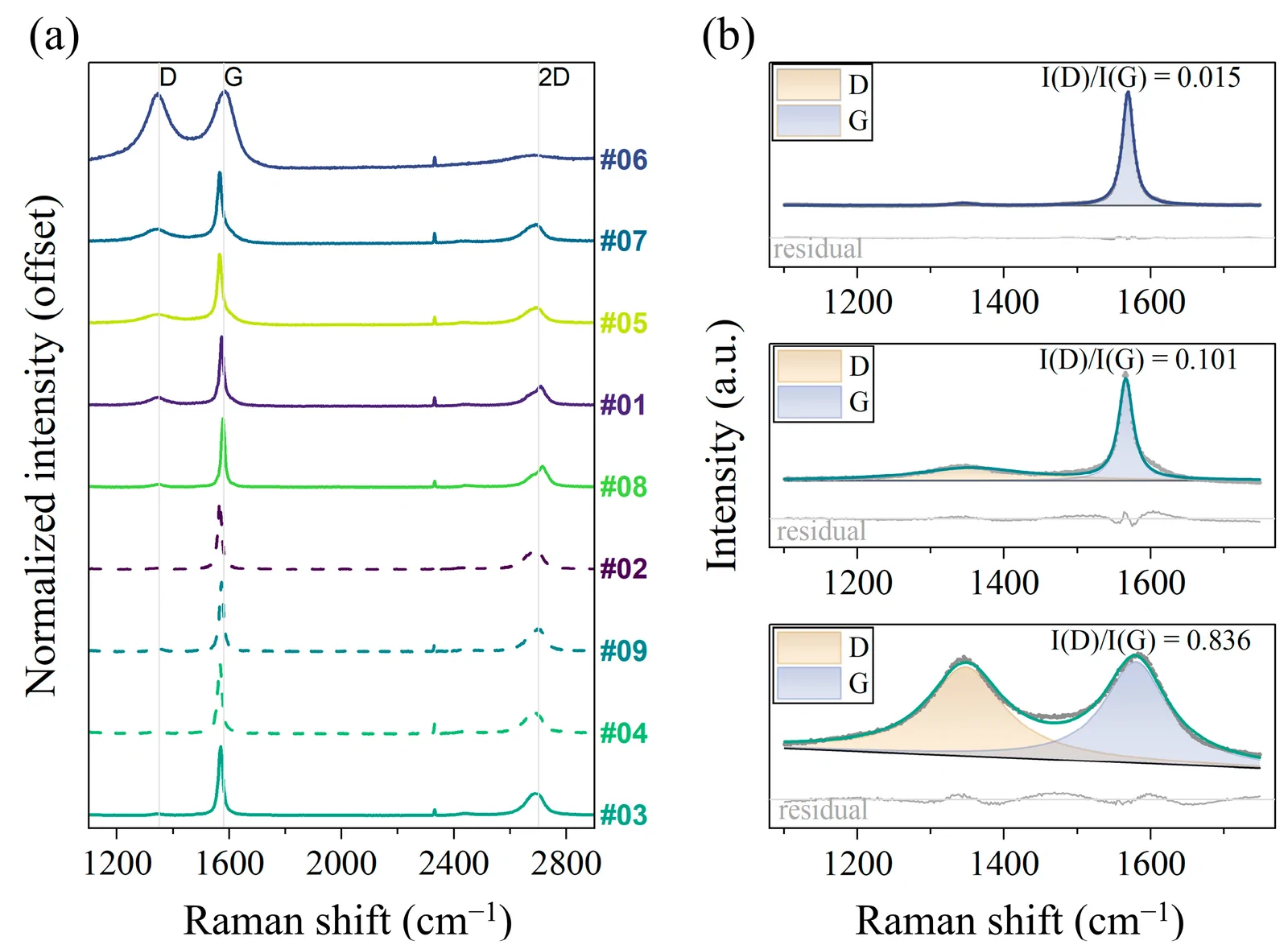
Raman spectra of the nine materials recorded at 532.04 nm: (**a**) full spectra, offset vertically, showing the D, G, and 2D bands; (**b**) Lorentzian fits with residuals for three representative samples, #03 (uncoated graphite), #05 (coated graphite), and #06 (carbonized). The set resolves into three tiers by I(D)/I(G): #06 alone at 0.836; the three coated graphites at 0.094–0.211; and the five uncoated graphites at 0.015–0.036. Each spectrum is the average of five independent positions.

Refitting of all 45 Raman spectra (Table 6, Figure 6; individual fits in Figure S4 and Table S2) resolves the sample set into three tiers: the carbonized sample #06 alone, with I(D)/I(G) = 0.836 and a G-band width of 101.9 cm^−1^; the three coated graphites, at 0.094–0.211; and the five uncoated graphites, at 0.015–0.036.

Nitrogen physisorption places eight of the nine materials within a narrow band, 0.55–1.82 m^2^ g^−1^, with the coated petroleum-derived grade #07 an order of magnitude above them at 9.92 m^2^ g^−1^ (Table 7); #07 is also the only sample with a measurable micropore volume, and the *C* constants of 77–429 confirm that the areas were obtained in a regime where the BET analysis is valid (Table S3).

**Figure 7:**
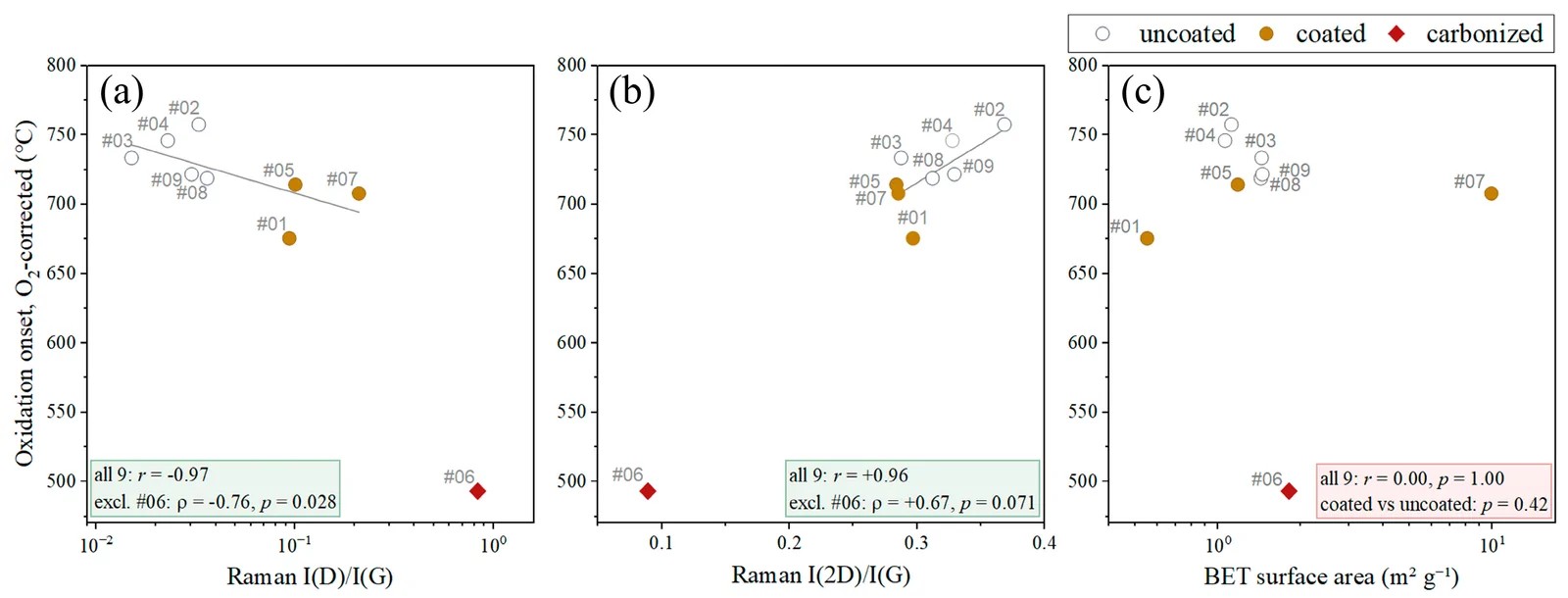
Structural and textural parameters tested against the O_2_-corrected oxidation onset: (**a**) Raman I(D)/I(G), logarithmic abscissa; (**b**) Raman I(2D)/I(G); (**c**) BET surface area, logarithmic abscissa. Coated materials are shown as filled symbols, uncoated as open symbols, and #06 as a diamond. Excluding #06, I(D)/I(G) retains a rank correlation with onset (ρ = −0.76, *p* = 0.028), whereas BET area shows none.

Across all 9 materials, the corrected oxidation onset correlated strongly with I(D)/I(G) (*r* = −0.97), but #06 dominated this correlation. With #06 excluded, the rank correlation survived (Spearman ρ = −0.76, *p* = 0.028), while the linear one did not (r = −0.54, *p* = 0.17); I(2D)/I(G) behaved similarly but more weakly (ρ = +0.67, *p* = 0.07), and the G-band width did not correlate (ρ = −0.48, *p* = 0.23). BET surface area showed no relationship to the corrected onset (*r* = 0.00, *p* = 1.00; ρ = −0.33, *p* = 0.38; Figure 7).

The scatter on #01 is notably larger than on any other sample (SD 0.118, mean 0.094; individual measurements 0.007–0.293), indicating heterogeneous coating coverage on the natural graphite rather than measurement error, since the five spectra were acquired under identical conditions.

## 4. Discussion

### 4.1. Residual Oxygen in Evolved-Gas Analysis of Graphite

The stoichiometric consumption of oxygen shows that, in these measurements, the CO_2_ evolved from graphite at high temperature is an oxidation product. Graphite is particularly susceptible because its inventory of surface oxygen groups is small after heat treatment at 3000 °C [12,31], so even trace residual oxygen supplies more CO_2_ than desorption does. Air ingress into the furnace of this type of hyphenated system has been noted before [34], and here it increased over consecutive runs on the same day.

Three practical consequences follow for evolved-gas studies of carbon materials under nominally inert gas. First, extensive quantities such as CO_2_ yield and mass loss measure the oxygen supplied and cannot be compared between runs unless the residual oxygen is matched. Second, normalized onsets carry information about the material but also depend on the oxygen level, with an apparent reaction order below unity [32,33]; here, a 2.7-fold change in oxygen shifted the onset by 25 °C, comparable to the material effects of interest. Third, the problem is readily diagnosed by comparing the O_2_ signal with inert references such as Ar or N_2_, which a time-of-flight analyzer records in every spectrum; in this study, we could diagnose it retrospectively from full-range spectra recorded without this in mind. We recommend that residual oxygen be reported alongside evolved-gas data for carbons and that comparisons be restricted to, or corrected for, matched oxygen levels.

### 4.2. Coating: Common Reactivity Effect, Precursor-Specific Texture

The two coatings examined here act in the same direction on the quantities that reflect surface reactivity—they raise Raman disorder by about sevenfold and lower the oxidation onset—but in opposite directions on texture. The coal-tar-derived coating fills and smooths the surface, lowering the BET area; the petroleum-derived coating deposits a microporous, hydrophilic layer, raising the BET area almost sevenfold and increasing water uptake from ambient air by two orders of magnitude.

The microporosity of #07 most likely originates in the coating precursor rather than in the base graphite, whose texture (#09) is unremarkable. A pitch layer carbonized on a graphite surface develops porosity as its volatile fraction escapes, and the amount of volatile matter lost depends on the pitch’s coking value: coal-tar pitch is more aromatic, passes through mesophase, and densifies to a more compact carbon, whereas petroleum pitch is comparatively aliphatic, loses a larger mass fraction on carbonization, and leaves a more open residue [9,10,11,41]. The high C constant, narrow mean pore width, and 126-fold increase in physisorbed water are consistent with an incompletely densified amorphous overlayer on #07 and a filled, densified one on #05. We note that this interpretation is based on the texture of the finished materials; the coking values and carbonization conditions used by the suppliers are not available to us, and confirming it would require coating the same base graphite with pitches of known coking value.

The divergence between texture and reactivity reflects the distinction between total and active surface area [17,18,19] in the context of carbon oxidation reactivity, where this distinction was first formulated. Oxidation proceeds at edge and defect sites, which contribute little to the nitrogen-accessible area of graphite [42,43]; a coating that increases the total area severalfold therefore need not increase reactivity more than one that decreases it, and in this case it did not. The active surface area was not measured in this work—it requires chemisorption of oxygen at 200–300 °C followed by temperature-programmed desorption of the resulting CO and CO_2_, which the present instrument configuration cannot perform because *m*/*z* 28 is obscured by the nitrogen background—so the evidence offered here for decoupling is indirect and rests on two observations. Across the eight graphitized materials, the BET area spans a factor of 18, from 0.55 to 9.92 m^2^ g^−1^, while the corrected oxidation onset spans 82 °C with no correlation between them (r = 0.00, Figure 7). Within the two coated pairs, one coating lowers the BET area by 18%, and the other raises it by 581%; both shift the onset in the same direction by a similar amount and raise I(D)/I(G) by about the same factor of seven. Direct determination of the active surface area on these nine materials would convert this argument from indirect to direct and is the principal extension we propose. For specifying coated anode grades, this has two implications. BET area does not indicate how much a coating changes surface reactivity, and grades from different precursors that are similar in reactivity may still differ greatly in texture and moisture uptake, which matter for electrode processing and the first cycle.

### 4.3. Precursor-Specific Behavior

Coal-tar-derived graphites oxidized later than petroleum-derived ones in every available precursor-matched comparison, including one prepared in-house by an identical route, even though their Raman parameters overlapped. This complements our earlier finding that XRD, Raman spectroscopy, and TEM do not distinguish between graphites of these two origins [8]: the oxidation onset is sensitive to a surface property that bulk parameters do not capture. The present data cannot identify the origin of this difference. Differences in edge structure inherited from mesophase development [10] are one possibility, and heteroatoms, present in coal tar but not in pyrolysis fuel oil [8], are another, although no sulfur- or nitrogen-bearing species were detected in the evolved gases.

The coated natural graphite behaves differently from both coated artificial grades. The wide scatter in its Raman ratios, the narrow D band, and the retained crystal faces point to incomplete coverage, and its earlier oxidation is consistent with part of the graphite surface remaining exposed. That even this partial coating places #01 within the Raman disorder range of coated materials suggests that Raman mapping, rather than a single-point measurement, is the appropriate way to assess coating uniformity on natural graphite.

### 4.4. Heat-Treatment History

The largest and most robust effect in the data set is heat-treatment temperature, which controls the degree of structural order in carbon [7]. The carbonized coke oxidizes at more than 180 °C earlier than any graphitized material, and graphitization of the same coke raises the onset by 225 °C at the same oxygen level. Differences of this magnitude dwarf those associated with precursor, texture, or coating, so compare precursor and coating effects between materials with matched heat-treatment history, as the paired design here allows.

### 4.5. Relation to Electrode Performance

We made no electrochemical measurements in this work; we characterized the nine materials as powders, and the study is a materials-characterization study rather than a synthesis or cell study. The connection to cell behavior is therefore an inference, and we state it as such.

This inference rests on the same sites being involved. Molecular oxygen oxidation initiates at prismatic edges and defect sites rather than on basal planes [17,18,19,42,43], and these are also the sites where electrolyte reduction begins and the solid–electrolyte interphase nucleates on the first charge [20,22,23,24,25,26]. A material that begins to oxidize at a lower temperature therefore presents a larger or more reactive population of such sites and is expected to consume more lithium irreversibly in the first cycle. On that reading, the 225 °C separation between the carbonized and graphitized forms of the same coke corresponds to the well-documented loss of initial Coulombic efficiency of under-graphitized carbon, whereas the 6–27 °C precursor difference is a far smaller effect that would be correspondingly harder to resolve electrochemically.

Two observations in the present data bear directly on how coated grades are specified. First, BET area, the parameter used industrially for this purpose, did not predict oxidation onset in any comparison, so it does not reflect the surface property that governs first-cycle loss; a grade selected on BET area alone may differ in reactivity from one selected on the same number. Second, the two coatings examined here are equivalent in reactivity but not in texture or moisture uptake: the petroleum-derived coated grade #07 held 126 times more physisorbed water than its uncoated counterpart, which matters for slurry preparation, electrode drying, and residual water reaching the cell, independently of any effect on the first cycle [6,41].

Testing the inference requires half-cell data on the same nine powders—first-cycle Coulombic efficiency and rate capability measured under a common protocol—compared with the corrected oxidation onsets reported here. Our earlier study of the in-house materials found that half-cell performance did not distinguish between graphites from the two precursor origins [8], consistent with reversible capacity being set by the graphitic interior, whereas the difference detected here resides at the surface. Whether first-cycle efficiency, a surface quantity, follows the oxidation onset remains to be established and is the subject of ongoing work.

### 4.6. Limitations

The principal limitation of this study is that we did not control residual oxygen. The correction applied to the onsets rests on a single replicate of a single material measured at two oxygen levels, and it assumes that the same sensitivity s applies to all nine samples. That assumption is neither verified here nor self-evidently true: s = nRT^2^/Ea depends on the apparent reaction order and the activation energy, both of which may differ between a graphitized surface and a coated or carbonized one. The corrections are also unevenly distributed. They are below 7 °C for every uncoated sample because those runs happened to fall near the reference oxygen level, but +26 °C for #05 and +44 °C for #07, comparable to or larger than the net coating effect remaining after correction (14–19 °C). The quantitative assessment of the coating effect is therefore conditional on the correction model, and we do not claim otherwise.

What can be stated without that reservation is set out in Table 3. The graphitization result is independent of *s* because the two runs were conducted at the same oxygen level. Two of the three precursor comparisons hold for any non-negative s, and the third holds for s up to 1.4 times the calibrated value. The direction of the coating effect holds up to 1.8 and 1.3 times the calibrated value in the two pairs, and is the same at the 5%, 10%, and 20% thresholds, but its magnitude should be regarded as uncertain. Verifying the assumption of a common sensitivity requires replicate measurements of at least one additional material—ideally a coated grade and the carbonized coke—at two known oxygen levels, which is the first of the additional experiments proposed below. The precursor comparisons involve at most three samples per group and are consistent in direction rather than statistically established. We could not quantify CO because nitrogen from the same air ingress dominates *m*/*z* 28, and the *m*/*z* 45/44 ratio (~0.08) reflects spillover from the intense *m*/*z* 44 peak rather than the ^13^C isotope, so it cannot confirm the *m*/*z* 44 assignment. We performed nitrogen physisorption after degassing at ambient temperature, and #04 showed a total surface area of 0.92 m^2^ to the analyzer, marginally below the practical threshold for nitrogen adsorption. Repeating the TGA–TOF MS measurements, with residual oxygen verified as negligible or deliberately set to a known level, would eliminate the need for correction and allow direct quantification of precursor and coating effects.

## 5. Conclusions

This work aimed to determine whether evolved-gas analysis by TGA–TOF MS resolves precursor- and coating-specific differences between graphite anode materials that bulk structural and textural parameters do not resolve. We addressed all four objectives set out in the Introduction; we met three without qualification, and we met one in direction but only conditionally in magnitude.

We met the first objective: establishing the origin of the high-temperature CO_2_. Full-range mass spectra showed that the CO_2_ evolved above 600 °C arose from oxidation of carbon by residual oxygen: O_2_ was consumed by 67–80%, while the inert reference ions changed by less than 13%, and the CO_2_ formed was proportional to the available oxygen (*r* = 0.9988), with no oxygen-independent component detectable. CO_2_ yield and mass loss are therefore properties of the measurement atmosphere rather than the material and were excluded from between-sample comparison, whereas the normalized oxidation onset remains a material property once corrected to a common oxygen level.

The second objective, quantifying the effect of the pitch-derived carbon coating, was met in direction but conditionally in magnitude. After correction, the coating lowered the oxidation onset by 19 °C for the coal-tar-derived material and by 14 °C for the petroleum-derived material in both commercial pairs, and raised I(D)/I(G) by about sevenfold in both. Its effect on texture was precursor-specific and opposite: the coal-tar-derived coating smoothed the surface and reduced the BET area by 18%, whereas the petroleum-derived coating deposited a microporous, hydrophilic layer that increased the BET area by 581%, contributed 30% of that area as micropores, and increased low-temperature water release 126-fold. The coating on the natural graphite was discontinuous, as shown by the scatter in its Raman ratios and the retained crystal faces.

The third objective was met. Coal-tar-derived graphites oxidized 6–27 °C later than petroleum-derived ones in all three precursor-matched comparisons, including a pair prepared in-house by an identical route, although the two families could not be distinguished by their Raman parameters. Graphitization of the carbonized petroleum coke raised the oxidation onset by 225 °C at the same oxygen level, and BET surface area did not predict the onset in any comparison.

The fourth objective, determining how far these conclusions depend on the oxygen correction, was met and defines the boundary of what the study establishes. The graphitization result is independent of the correction coefficient; two of the three precursor comparisons hold for any non-negative value of it and the third for values up to 1.4 times the calibrated one; the direction of the coating effect holds up to 1.3–1.8 times the calibrated value, but its magnitude does not, and should be treated as provisional.

Two applications follow. For laboratory practice, residual oxygen should be monitored against inert reference ions and reported alongside evolved-gas data for carbon materials, and comparisons restricted to, or corrected for, matched oxygen levels; a time-of-flight analyzer records the necessary channels in every spectrum at no additional cost, and the diagnosis reported here was possible only because it had done so. For anode specification and incoming quality control, this oxidation-onset measurement separates materials that BET area and Raman parameters do not, requires milligram quantities of powder, and needs no cell assembly, making it a candidate screening parameter for coated grades and for verifying precursor origin. Realizing that potential requires the two measurements identified in Section 4.6: replicate runs at known oxygen levels to establish that the correction is material-independent, and direct determination of the active surface area alongside half-cell first-cycle efficiency on the same powders.

## Figures and Tables

**Table 1:** Samples Identification.

No.	Sample	Precursor	Texture	Highest Heat Treatment	Coating	Origin
#01	GNA	Natural	—	—	Coated	Commercial
#02	GCT_iso	Coal tar	Isotropic	3000 °C	Uncoated	In-house [8]
#03	CCGA	Coal tar	Isotropic	n.d.	Uncoated	Commercial
#04	GCT_aniso	Coal tar	Anisotropic	3000 °C	Uncoated	In-house [8]
#05	CCGA_Co	Coal tar	Isotropic	n.d.	Coated	Commercial; coated form of #03
#06	CPFO_aniso	Petroleum	Anisotropic	1350 °C	Uncoated	In-house [8]
#07	PCGA_Co	Petroleum	Isotropic	n.d.	Coated	Commercial; coated form of #09
#08	GPFO_aniso	Petroleum	Anisotropic	3000 °C	Uncoated	In-house [8]; graphitized #06
#09	PCGA	Petroleum	Isotropic	n.d.	Uncoated	Commercial

Optical texture of the four commercial materials graphites (#03, #05, #07, and #09) is taken from the supplier specifications; —, not applicable; n.d., not disclosed by the supplier.

**Table 2:** Residual oxygen, onset temperatures and mass loss. Onsets are defined by the 10% criterion (Section 2.3).

No.	Sample	*m*_0_ (mg)	Residual O_2_ (10^3^ Counts)	O_2_ Consumed, 900–999 °C (%)	CO_2_ Onset, as Measured (°C)	CO_2_ Onset, O_2_ Corrected (°C)	Mass-Loss Onset (°C)	Mass Loss (%) ^a^
01	GNA	63.08	36.4	79.7	668.7	675.6	743.9	1.19
01 rep. ^b^	GNA	n.a.	13.6	72.9	643.5	675.6	n.a.	n.a.
02	GCT_iso	49.63	50.7	74.6	759.2	757.5	816.0	1.35
03	CCGA	50.59	44.3	75.7	731.9	733.7	792.2	1.11
04	GCT_aniso	43.68	47.6	74.0	745.9	745.9	806.5	1.44
05	CCGA_Co	70.80	16.9	76.4	687.8	714.3	764.6	0.39
06	CPFO_aniso	44.77	57.1	78.4	498.1	493.4	596.3	2.89
07	PCGA_Co	50.81	8.7	67.3	664.4	708.0	776.5	0.27
08	GPFO_aniso	39.45	57.5	72.2	723.7	718.8	789.8	2.06
09	PCGA	72.72	58.0	76.6	726.8	721.7	794.8	1.17

n.a., not available. ^a^ Mass loss over the complete program; it reflects the residual oxygen supplied and is not compared between samples. ^b^ Replicate of #01, measured on 24 July 2025 to calibrate the oxygen correction; its thermogravimetric record was not retained. Its corrected onset equals that of #01 by construction, since the pair defines the correction.

**Table 6:** Raman parameters (mean ± SD, *n* = 5 positions per sample).

No.	Sample	I(D)/I(G)	I(2D)/I(G)	G Position (cm^−1^)	G FWHM (cm^−1^)	D FWHM (cm^−1^)
01	GNA	0.094 ± 0.118	0.297 ± 0.021	1574.0	19.9 ± 5.9	52.6
02	GCT_iso	0.033 ± 0.016	0.369 ± 0.082	1571.1	18.3 ± 2.9	32.1
03	CCGA	0.015 ± 0.004	0.288 ± 0.040	1569.9	16.2 ± 2.0	28.3
04	GCT_aniso	0.023 ± 0.018	0.328 ± 0.091	1568.5	17.7 ± 2.5	26.5
05	CCGA_Co	0.101 ± 0.028	0.284 ± 0.033	1571.3	20.9 ± 3.0	155.6
06	CPFO_aniso	0.836 ± 0.033	0.089 ± 0.008	1584.4	101.9 ± 2.5	122.2
07	PCGA_Co	0.211 ± 0.031	0.286 ± 0.035	1568.4	27.4 ± 2.2	142.8
08	GPFO_aniso	0.036 ± 0.017	0.312 ± 0.042	1574.4	15.9 ± 2.0	35.1
09	PCGA	0.030 ± 0.012	0.329 ± 0.036	1570.5	17.5 ± 1.2	33.2

**Table 7:** Nitrogen physisorption results.

No.	Sample	Mass (g)	BET Area (m^2^ g^−1^)	t-Plot External Area (m^2^ g^−1^)	Micropore Volume (cm^3^ g^−1^)	Total Pore Volume (cm^3^ g^−1^)	Mean Pore Width (nm)	BET Constant, *C*
#01	GNA	2.100	0.553 ± 0.002	0.567	<0.0001	0.00175	12.6	248
#02	GCT_iso	1.217	1.121 ± 0.004	1.182	<0.0001	0.00274	9.8	77
#03	CCGA	1.474	1.447 ± 0.008	1.615	<0.0001	0.00639	17.7	95
#04	GCT_aniso	0.860	1.064 ± 0.002	0.993	<0.0001	0.00275	10.4	197
#05	CCGA_Co	1.009	1.182 ± 0.007	1.372	<0.0001	0.00415	14.0	105
#06	CPFO_aniso	0.965	1.821 ± 0.005	2.011	<0.0001	0.01073	23.6	261
#07	PCGA_Co	0.953	9.921 ± 0.036	6.963	0.00129	0.02083	8.4	429
#08	GPFO_aniso	1.007	1.435 ± 0.008	1.641	<0.0001	0.00648	18.1	223
#09	PCGA	1.458	1.457 ± 0.005	1.602	<0.0001	0.00628	17.3	224

## Data Availability

The original contributions presented in this study are included in the article and Supplementary Materials. The corresponding author can provide the raw thermogravimetric and full-range mass-spectrometric data files, the Raman spectra, and the nitrogen physisorption isotherms upon reasonable request. Further inquiries can be directed to the corresponding author.

## Supplementary Materials

The following supporting information can be downloaded at: https://www.mdpi.com/article/10.3390/ma19214550/s1. Figure S1: CO_2_ yield against gravimetric mass loss; Figure S2: low-temperature water release; Figure S3: nitrogen adsorption–desorption isotherms; Figure S4: Lorentzian fits to all 45 Raman spectra; Table S1: thermogravimetric–mass spectrometric run conditions; Table S2: Lorentzian fit parameters of the 45 Raman spectra; Table S3: nitrogen physisorption quality metrics; original uncropped scanning electron micrographs of the nine materials.
