# Supplementary material for: Separating Material Reactivity from Residual Oxygen in TGA–TOF MS of Natural and Artificial Graphite Anodes
Source: Materials (Basel). 2026 Oct 28;19(21):4550. doi: 10.3390/ma19214550 (PMC13764791; doi:10.3390/ma19214550)
Supplement: Supplementary file 1 [file materials-19-04550-s001.zip › materials-4638120-supplementary.pdf]

Supplementary Materials

Separating Material Reactivity from Residual Oxygen in TGA–TOF MS of Natural and Artificial Graphite Anodes

Hee-Jin Yoo [1,2], Minsun Kim [1], Young Hwan Kim [3], Woo Jin Jeon [4], Byung Gwun Jin [4], Jooyeon Oh [4], Hyun Sik Kim [4], Yangsun Kim [4], Young-Kwan Kim [5], Han Bin Oh [2] and Kun Cho [1,6,*]

[1] Biopharmaceutical Mass Spectrometry Research Unit, Korea Basic Science Institute, Cheongju 28119, Republic of Korea; rhfvm2@kbsi.re.kr (H.-J.Y.); kminsun@kbsi.re.kr (M.K.)
[2] Department of Chemistry, Sogang University, Seoul 04100, Republic of Korea
[3] Division of Materials Science, Korea Basic Science Institute, Daejeon 34133, Republic of Korea; yhkim@kbsi.re.kr
[4] ASTA Corporation, 145 Gwanggyo-ro, Yeongtong-gu, Suwon 16229, Republic of Korea; woojin.jeon@astams.com (W.J.J.); byunggwun.jin@astams.com (B.G.J.); jyoh@astams.com (J.O.); hskim@astams.com (H.S.K.); yangskim@astams.com (Y.K.)
[5] Department of Chemistry, Dongguk University, Seoul 04620, Republic of Korea; kimyk@dongguk.edu
[6] Department of Bio-Analytical Science, University of Science and Technology, Daejeon 34113, Republic of Korea
* Correspondence: chokun@kbsi.re.kr; Tel.: +82-43-240-5143; Fax: +82-43-240-5159

Table S1. Thermogravimetric–mass spectrometric run conditions. All runs used helium at 70 mL min^{-1}, an initial hold at the starting temperature, a ramp of 20 min^{-1} to 1000 ℃ followed by a 10 min hold, a transfer line at 250 ℃, and electron-impact ionization at 80 eV. Samples were measured between 24 and 31 July 2025. Full spectra over *m/z* 1-300 were recorded for every run and used for all analyses. The #01 replicate was measured to calibrate the oxygen correction (Section 2.3).

No	Sample	Acquisition date	Sample mass (mg)	Mass loss, ramp (%)	Mass loss, isothermal hold (%)	Isothermal fraction (%)
#01	GNA	2025-07-31	63.078	0.814	0.378	32.0
#02	GCT_iso	2025-07-25	49.629	0.787	0.562	42.0
#03	CCGA	2025-07-25	50.587	0.669	0.442	40.0
#04	GCT_aniso	2025-07-24	43.678	0.868	0.576	40.0
#05	CCGA_Co	2025-07-25	70.798	0.256	0.13	34.0
#06	CPFO_aniso	2025-07-25	44.774	2.103	0.782	27.0
#07	PCGA_Co	2025-07-28	50.808	0.181	0.091	33.0
#08	GPFO_aniso	2025-07-24	39.453	1.251	0.806	39.0
#09	PCGA	2025-07-28	72.723	0.713	0.46	39.0

Table S2. Lorentzian fit parameters for each of the 45 Raman spectra, five independent positions per sample. Spectra were baseline-corrected with a linear function over 1100-1750 cm^{-1} and 2550-2850 cm^{-1} before fitting. The five positions in sample #01 span I(D)/I(G) values from 0.007 to 0.293, an order of magnitude wider than in any other sample.

Sample	Code	Position	I(D)/I(G)	I(2D)/I(G)	D position (cm-1)	G position (cm-1)	D FWHM (cm-1)	G FWHM (cm-1)
**#01**	GNA	1	0.1109	0.2723	1345.8	1573	78	18.2
**#01**	GNA	2	0.0248	0.2952	1340.2	1567.4	40	19.9
**#01**	GNA	3	0.0068	0.2805	1349.4	1576.1	16.3	14.4
**#01**	GNA	4	0.2933	0.3217	1350.5	1578.5	93.5	29.8
**#01**	GNA	5	0.0331	0.315	1346.7	1575.3	35.3	17.4
**#02**	GCT_iso	1	0.0152	0.2841	1341.9	1566.3	28.4	16.5
**#02**	GCT_iso	2	0.0459	0.436	1345.1	1568.1	25.9	23
**#02**	GCT_iso	3	0.0449	0.4237	1344.4	1572.7	37.4	18.8
**#02**	GCT_iso	4	0.0413	0.4239	1346.3	1575	37.5	17.9
**#02**	GCT_iso	5	0.0164	0.2748	1342.1	1573.3	31.1	15.4
**#03**	CCGA	1	0.0185	0.3394	1344.8	1569.1	33	18.3
**#03**	CCGA	2	0.0156	0.2492	1344.8	1570.3	27.8	14.5
**#03**	CCGA	3	0.0094	0.259	1347.8	1571.4	20.2	14.5
**#03**	CCGA	4	0.0187	0.3223	1344.7	1567.5	33.6	18.5
**#03**	CCGA	5	0.0132	0.2687	1347.7	1571.1	26.8	15.3
**#04**	GCT_aniso	1	0.0271	0.306	1341.5	1566.3	31.6	18.9
**#04**	GCT_aniso	2	0.0324	0.4065	1352.1	1578.9	31.9	17.7
**#04**	GCT_aniso	3	0.0462	0.4356	1344.2	1570.3	37.8	21.1
**#04**	GCT_aniso	4	0.0046	0.2697	1342	1562.5	19.6	15.9
**#04**	GCT_aniso	5	0.0044	0.2218	1347.2	1564.6	11.8	14.8
**#05**	CCGA_Co	1	0.129	0.2589	1354.6	1566.3	184.3	23.6
**#05**	CCGA_Co	2	0.0727	0.2759	1356.5	1574.6	140.4	17.2
**#05**	CCGA_Co	3	0.0798	0.2557	1356.5	1571.6	148.7	18.7
**#05**	CCGA_Co	4	0.092	0.3378	1354.5	1573.7	136.9	20.6
**#05**	CCGA_Co	5	0.1311	0.2902	1354.8	1570.3	167.5	24.2
**#06**	CPFO_aniso	1	0.8735	0.0877	1347.4	1579.6	124.4	104.1
**#06**	CPFO_aniso	2	0.8225	0.0925	1352.8	1586.6	116.9	99.7
**#06**	CPFO_aniso	3	0.8512	0.099	1353.3	1588.3	114.5	99.6
**#06**	CPFO_aniso	4	0.8475	0.0896	1349.7	1583.3	122.4	101.2
**#06**	CPFO_aniso	5	0.7872	0.077	1352	1584.1	132.9	105.1
**#07**	PCGA_Co	1	0.182	0.2769	1345.1	1566.2	131.5	25.7
**#07**	PCGA_Co	2	0.2131	0.2803	1348.4	1567.3	159.6	28.7
**#07**	PCGA_Co	3	0.2287	0.3441	1347.1	1569.3	129.3	30.2
**#07**	PCGA_Co	4	0.2515	0.2781	1350.8	1573.8	144.9	27.7
**#07**	PCGA_Co	5	0.178	0.2477	1346.3	1565.7	148.8	24.9
**#08**	GPFO_aniso	1	0.0377	0.2873	1346.2	1577.7	38.5	14.7
**#08**	GPFO_aniso	2	0.0256	0.2772	1341.2	1569.6	29.7	16.2

**#08**	GPFO_aniso	3	0.0347	0.3003	1346.9	1577.3	36.4	14.8
**#08**	GPFO_aniso	4	0.0642	0.3832	1342.3	1570.9	38.3	19.2
**#08**	GPFO_aniso	5	0.0189	0.3125	1347.5	1576.4	32.4	14.4
**#09**	PCGA	1	0.0489	0.3424	1345.4	1572.2	30.7	17.2
**#09**	PCGA	2	0.0351	0.3296	1345.9	1573.9	37	17.2
**#09**	PCGA	3	0.0184	0.2914	1341.9	1566.3	31.6	16.3
**#09**	PCGA	4	0.0276	0.382	1350.4	1577.1	33.5	17.4
**#09**	PCGA	5	0.021	0.3005	1338.7	1563.1	33.2	19.5

Table S3. Nitrogen physisorption quality metrics. BET areas were calculated over P/P_0 = 0.057-0.180 with a nitrogen cross-sectional area of 0.1620 nm^2. C constants above roughly 200 indicate strong adsorbate–adsorbent interaction; sample #07, with C = 429 and the only positive t-plot micropore volume in the set (Table 7), is interpreted as genuinely microporous. Sample #04 had a total surface area of 0.92 m^2, marginally below the practical threshold for nitrogen adsorption.

No	Sample	Sample mass (g)	BET area (m^2/g)	BET C constant	BET correlation coefficient	Total surface area presented (m^2)	Below 1 m^2 threshold
**#01**	GNA	2.1001	0.5529	247.7	0.9999483	1.161	no
**#02**	GCT_iso	1.2171	1.1212	77.4	0.9999301	1.365	no
**#03**	CCGA	1.4736	1.4469	94.9	0.9999022	2.132	no
**#04**	GCT_aniso	0.8599	1.0635	197.4	0.9999911	0.915	yes
**#05**	CCGA_Co	1.0088	1.1822	104.5	0.9999107	1.193	no
**#06**	CPFO_aniso	0.9647	1.8207	261.3	0.9999786	1.756	no
**#07**	PCGA_Co	0.9533	9.9213	428.9	0.9999604	9.458	no
**#08**	GPFO_aniso	1.0072	1.4351	222.7	0.9999324	1.445	no
**#09**	PCGA	1.4576	1.4569	217.1	0.9999752	2.124	no

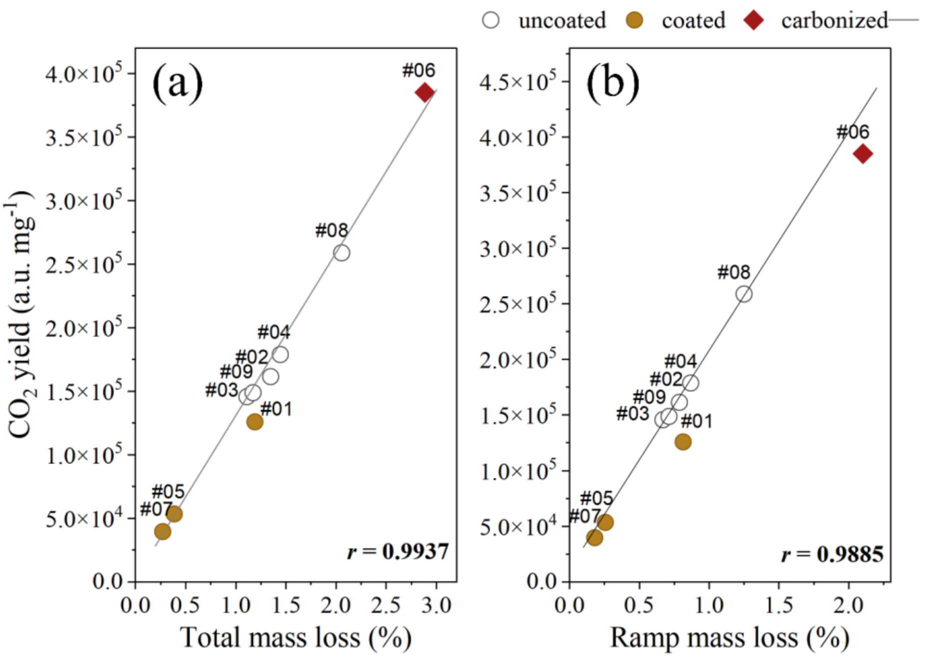

Figure S1. Mass-normalized integrated CO_2 yield plotted against gravimetric mass loss for the nine materials: (a) against the total mass loss over the complete program (r = +0.994); (b) against the ramp segment alone (r = +0.989). Both quantities record the carbon oxidized by residual oxygen (Section 3.2), which is why they agree; neither is compared between samples.

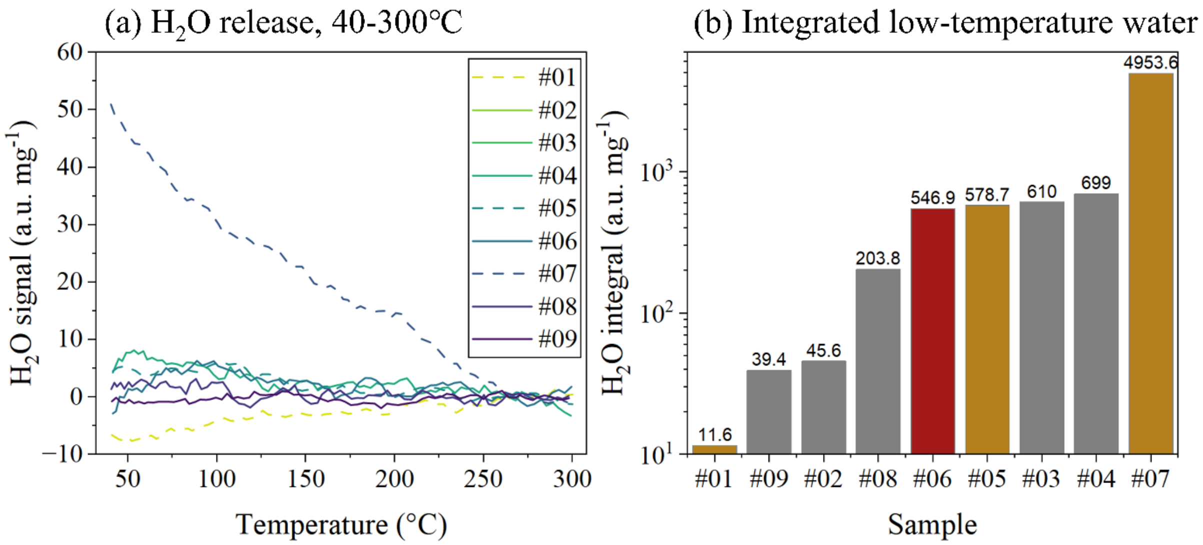

Figure S2. H_2O (m/z 18) ion current below 300 °C: (a) mass-normalized ion current between 40 °C and 300 °C, with the startup transient below 40 °C excluded; (b) the corresponding integrals, ordered by magnitude. Eight of the nine materials release little low-temperature water, supporting the choice of ambient-temperature degassing described in Section 2.5. Sample #07 is the exception, releasing 7.1 times more than the next-highest material. Since #07 also has by far the largest BET surface area, any residual water remaining after ambient-temperature degassing would underestimate its surface area, which would work against rather than in favor of the difference reported in Section 3.4.

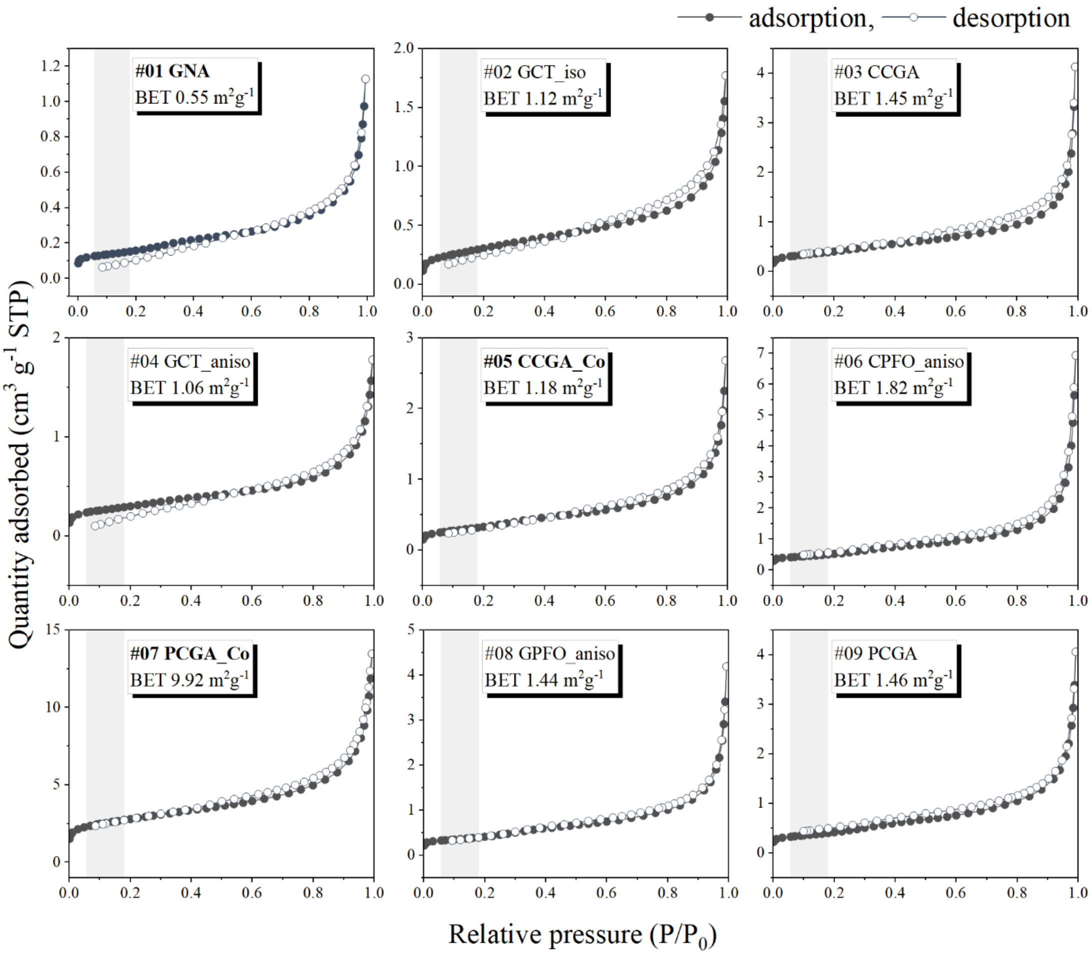

Figure S3. Nitrogen adsorption–desorption isotherms at 77.3 K for the nine materials, with the relative pressure range used for the BET calculation shaded. All isotherms are of type II. Sample #07 alone shows appreciable low-pressure uptake, consistent with its positive t-plot micropore volume and its BET C constant of 429; the remaining eight give C constants between 77 and 261 and no measurable microporosity.

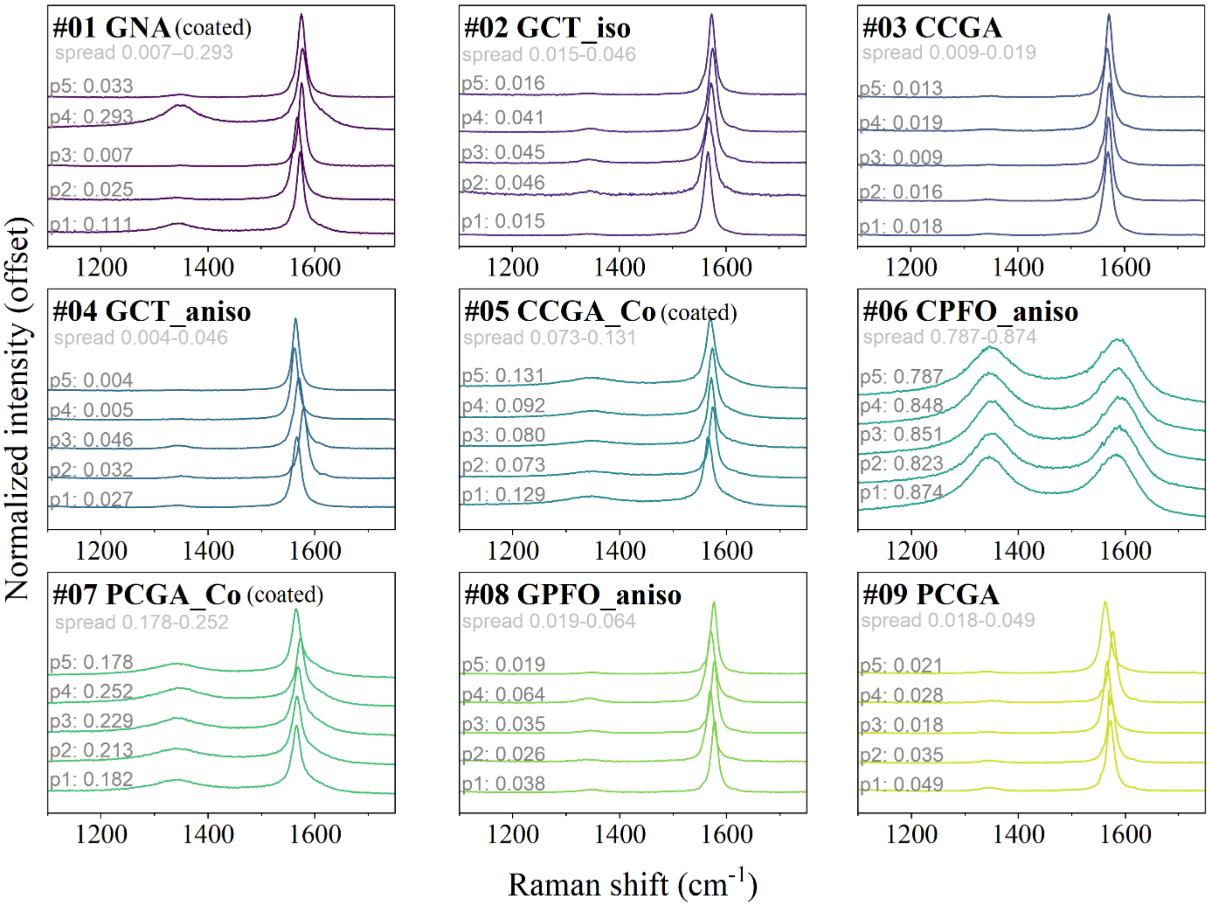

Figure S4. The five Raman spectra recorded at independent positions on each sample, offset vertically and normalized to their own maxima. The number beside each trace is the I(D)/I(G) ratio obtained by fitting that spectrum with two Lorentzian components on a linear baseline, and the range of the five values is given under each sample name. The five positions measured on #01 span 0.007 to 0.293, an order of magnitude wider than any other sample, which is the basis for the conclusion in Section 4.3 that its coating is discontinuous.
